# Model-Based Decomposition of Low-Altitude Infrared Radiance Using Dual-Band MWIR Observations

**DOI:** 10.3390/s26216959

**Published:** 2026-11-04

**Authors:** Jeong-Hyeon Moon, Jangbeom Lee, Bongjun Choi, Heeduk Park

**Affiliations:** Hanwha Systems Co., Ltd., 188 Pangyoyeok-ro, Bundang-gu, Seongnam-si 13524, Gyeonggi-do, Republic of Korea; jb87.lee@hanwha.com (J.L.); bj19.choi@hanwha.com (B.C.); heeduk.park@hanwha.com (H.P.)

**Keywords:** low-altitude slant path, mid-wave infrared dual band, path radiance, solar scattering, radiometric calibration, surface temperature retrieval

## Abstract

Infrared image radiance combines surface and atmospheric thermal emission with solar contributions, making agreement in total radiance insufficient to assess individual components. This study presents a model-based decomposition method using two mid-wave infrared (MWIR) bands with contrasting atmospheric absorption and solar sensitivity. Viewing geometry recovered from imagery and terrain data is combined with meteorological inputs. Ground-target temperatures retrieved from the longer-wavelength band are held fixed in shorter-wavelength predictions, without further target fitting. Observations on two days test these predictions across different atmospheric and surface contributions. For forests at 280 and 1080 m, spanning modeled surface-dominated and path-dominated conditions, shorter-band radiance predicted using longer-band temperatures differs from observations by at most 0.056 W m^−2^ sr^−1^ (maximum absolute residual across 12 comparisons at six times). Independently retrieved long-wave infrared forest temperatures agree with MWIR estimates within 2.6 °C. In two comparison frames, using observation-constrained atmospheric inputs reduces the root-mean-square (RMS) difference between observed and predicted longer-band sky radiance to 1/10.9 and 1/15.4 of the values obtained with an unmodified standard atmosphere, with the same offset retained across profiles within each frame. Greenhouse observations extend the analysis to diffuse solar reflection and localized brightening consistent with specular reflection. These results demonstrate how cross-band comparisons can assess the modeled radiance composition of field imagery beyond agreement in total signal.

## 1. Introduction

Low-altitude infrared (IR) surveillance must accommodate diverse aerial targets, including unmanned aerial vehicles and low-flying missiles. These targets differ in size, speed, material, and thermal signature, placing different demands on detection systems. IR band selection therefore needs to account for this diversity and the conditions under which targets are observed, rather than relying on a single representative target signature.

For surveillance over a broad area, a wide field of view allows a larger region to be monitored in a single image. However, for a fixed detector array, wider coverage increases the angular extent represented by each pixel, or instantaneous field of view (IFOV). Distant small targets may therefore occupy only a few pixels or less, providing limited shape information for detection [1,2]. Under these conditions, the radiance contrast between a target and its background becomes particularly important. This contrast depends on the spectral band and the radiance contributions of both the target and its background.

Observed radiance combines components with different spectral and geometric dependencies (Figure 1). The sky background contains thermal path emission and scattered sunlight. Ground-target emission and reflected sky and solar radiation, including specular glint, are attenuated along the viewing path and combined with atmospheric radiance at the sensor. Temperature, material properties, illumination, range, and atmospheric conditions determine the relative contributions.

Radiative-transfer models calculate individual radiance contributions, whereas a camera records their combined signal [3]. Comparing the two therefore requires converting camera output to radiance and representing the actual viewing paths and atmospheric conditions in the calculations. Radiometric calibration, viewing directions and target ranges, and meteorological information provide the necessary inputs [4]. With these inputs, comparisons across viewing direction, range, and spectral band can assess how consistently the calculated components account for the signals recorded in field imagery.

Previous studies have linked models and observations in different settings. Satellite vicarious calibration combines ground references, atmospheric information, and known geometry to evaluate sensor response along downward-looking paths [5,6,7], while ground-based infrared all-sky imaging samples atmospheric radiance across sky directions [8,9]. In field comparisons more directly concerned with atmospheric and surface contributions, Grimming et al. constructed atmospheric profiles from contemporaneous observations and evaluated predicted sky brightness temperatures [4]. Leslie et al. modeled power-line emission and reflection together with background radiance and compared the predictions with mid-wave and long-wave infrared (MWIR and LWIR) measurements [3]. These studies provide precedents for relating atmospheric and surface radiance components to field observations.

For low-altitude surveillance, the viewing path can extend for several kilometers through the lower atmosphere, requiring both attenuation of surface radiation and accumulated atmospheric emission to be considered. Simulation studies of infrared band selection account for these effects when predicting target-to-background contrast and detection range [10,11]. Field observations can test whether the components underlying those predictions account for the recorded signals. In particular, observing the same scene in bands with contrasting atmospheric absorption and solar sensitivity allows evaluation beyond agreement with total radiance in a single band: a consistent component model should also account for the differences between bands across viewing directions and surface types.

This study presents a model-based procedure for decomposing sky and ground-target radiance along low-altitude slant paths and evaluating the component interpretation through dual-band MWIR observations. The observation bands lie on opposite sides of the CO_2_ absorption feature near 4.3 μm; the shorter-wavelength band is designated BLUE and the longer-wavelength band RED. RED has stronger atmospheric absorption and a weaker solar contribution, whereas BLUE retains greater transmittance and a stronger solar contribution. For ground targets, surface temperature is retrieved from RED with specified emissivity and held fixed in the BLUE prediction, including solar reflection. No additional target parameter is adjusted to match BLUE, so the second band provides a check of the component model rather than a separate temperature fit. Radiometric calibration, viewing geometry recovered from images, satellite imagery, and digital elevation data, and contemporaneous meteorological inputs establish the measurements and observation paths needed for these comparisons.

The component model is evaluated for sky and ground targets using observations from two days with contrasting atmospheric conditions. Near and far forests test the cross-band prediction under modeled surface-dominated and path-dominated conditions, while the near greenhouse extends the comparison to a surface with a larger diffuse solar-reflection contribution. Auxiliary LWIR observations provide a separate temperature cross-check for the far forest. Sky comparisons examine radiance variation with elevation and solar-relative viewing direction. The observation-based atmospheric inputs reduce RED sky-radiance root-mean-square (RMS) error by a factor of approximately 11–15 compared with predictions using an unmodified standard atmosphere, under the same offset correction in the two comparison frames. Localized brightening of distant greenhouse roofs is examined separately for consistency with solar reflection using reflection geometry and an idealized radiance reference.

## 2. Theory

This section describes the radiance components reaching the sensor and their dependence on atmospheric paths, illumination, and surface properties. The observation geometry used in these calculations is estimated as described in Section 3.2.

### 2.1. Composition of the Observed Radiance

Radiance reaching the sensor combines surface contributions attenuated by the atmosphere with radiation added along the viewing path. Using band-effective surface properties and atmospheric transmittance, these contributions are represented by [12]

(1) $$L_{k}=\tau_{k}\left(R\right)[\underset{\text{self-emission}}{\underset{︸}{\varepsilon_{k}B_{k}\left(T_{s}\right)}}+\underset{\mathrm{thermal}\;\mathrm{sky}\;\mathrm{reflection}}{\underset{︸}{L_{k}^{\mathrm{ref},\mathrm{sky}}}}+\underset{\mathrm{solar}\;\mathrm{reflection}}{\underset{︸}{L_{k}^{\mathrm{ref},\mathrm{sol}}}}+\underset{\mathrm{transmitted}\;\mathrm{background}}{\underset{︸}{t_{o,k}L_{k}^{\mathrm{bg}}}}]+\underset{\mathrm{atmospheric}\;\mathrm{radiance}}{\underset{︸}{L_{k}^{\mathrm{atm}}}},$$

Here, *k* identifies the sensor band, *R* is target range, and $\tau_{k}\left(R\right)$ is the atmospheric transmittance from target to sensor. The surface emits with band emissivity $\varepsilon_{k}$ at temperature $T_{s}$; $B_{k}\left(T_{s}\right)$ is the corresponding blackbody radiance integrated over the sensor response. The target’s own transmittance $t_{o,k}$ allows background radiance $L_{k}^{\mathrm{bg}}$, incident from behind the target, to pass through it. This term vanishes for an opaque target. Band radiances are weighted by the spectral response $S_{k}\left(\lambda \right)$; for example, $B_{k}\left(T\right)=\int B_{\lambda }\left(T\right)S_{k}\left(\lambda \right)\;d\lambda$.

The reflected terms distinguish atmospheric thermal radiation from sunlight. For an opaque Lambertian surface, the band reflectance is $\rho_{k}=1-\varepsilon_{k}$, and reflected thermal sky radiance is

$$L_{k}^{\mathrm{ref},\mathrm{sky}}=\left(1-\varepsilon_{k}\right)L_{k}^{\mathrm{sky}↓}.$$

The quantity $L_{k}^{\mathrm{sky}↓}$ is the incident thermal sky irradiance divided by π, an equivalent radiance for diffuse reflection rather than sky radiance in a single viewing direction. Direct sunlight and sunlight scattered by the atmosphere before reaching the surface instead supply the solar-reflection term, described in Section 2.1.3.

The atmospheric term is $L_{k}^{\mathrm{atm}}=L_{k}^{\mathrm{path}}+L_{k}^{\mathrm{scat}}$, where $L_{k}^{\mathrm{path}}$ is thermal radiation emitted along the path and $L_{k}^{\mathrm{scat}}$ is sunlight scattered into the viewing direction by the atmosphere. Unlike surface-reflected sunlight, the latter reaches the sensor without a subsequent surface reflection. For the sky regions analyzed here, these atmospheric components form the scene radiance; instrumental glare is added separately in Section 3.4.

#### 2.1.1. Thermal Path Radiance Along a Slant Path

Thermal path radiance is the accumulated emission from the atmosphere, attenuated between each emitting location and the sensor. Neglecting scattering, this contribution at wavelength λ follows the Schwarzschild integral [13]:

(2) $$L_{\lambda }^{\mathrm{path}}=\int_{0}^{R}\kappa_{\lambda }\left(s\right)\;B_{\lambda }\left(T\left(s\right)\right)\;e^{-\delta_{\lambda }\left(0,s\right)}\;ds,$$

Distance *s* is measured from the sensor; $\kappa_{\lambda }\left(s\right)$ is the absorption coefficient, and T(s) is local air temperature. The absorption optical depth $\delta_{\lambda }\left(0,s\right)=\int_{0}^{s}\kappa_{\lambda }\left(u\right)\;du$ gives the transmission factor $e^{-\delta_{\lambda }\left(0,s\right)}$ between each emitting location and the sensor. For a path at a uniform temperature $T_{\mathrm{path}}$, the band-effective thermal path radiance can be approximated as [12]

$$L_{k}^{\mathrm{path}}\approx \left[1-\tau_{k}\left(R\right)\right]B_{k}\left(T_{\mathrm{path}}\right).$$

With $\tau_{k}\left(R\right)$ representing absorption-only transmittance, $1-\tau_{k}\left(R\right)$ acts as an effective path emissivity under these conditions. As $\tau_{k}\left(R\right)$ approaches zero, the thermal path radiance approaches the blackbody radiance $B_{k}\left(T_{\mathrm{path}}\right)$ at the atmospheric temperature. This approximation underlies the ground-target model in Section 3.5; the sky calculation retains the vertical temperature structure.

For sky observations, elevation changes the path length through each atmospheric layer and hence the accumulated thermal path radiance. Approximating the atmosphere as horizontally uniform, parallel layers, the path through a layer of thickness dz has length ds=dz/sin(el), where el>0 is viewing elevation. Lower elevations therefore lengthen the path through the warm lower atmosphere. In strongly absorbing bands, near-horizon thermal radiance approaches the blackbody radiance of near-surface air. A more transparent band samples farther into the colder upper atmosphere and has a darker thermal sky background [13,14]. These differences motivate comparisons across elevation as well as band.

Along a ground-target path, increasing atmospheric attenuation reduces the surface contribution while path emission becomes more prominent. The apparent target radiance approaches the atmospheric value, reducing target-to-background contrast [12].

#### 2.1.2. Solar Scattering

Solar scattering adds a sunlight-derived component to the thermal sky background. Molecules and aerosols redirect sunlight into the viewing direction; molecular scattering decreases rapidly with wavelength, while aerosols can concentrate scattered light near the sun’s apparent position [13,14].

In a horizontally uniform atmosphere, thermal path radiance varies with viewing elevation, whereas solar scattering also depends on the viewing azimuth relative to the sun. These different angular dependencies provide a basis for comparing the predicted thermal and solar components. Their separate radiative-transfer calculations are described in Section 3.3.

#### 2.1.3. Surface Reflection of Solar Radiation

Surface reflection redirects incident sunlight toward the sensor. Diffuse reflection distributes it over outgoing directions, whereas specular reflection concentrates it near the mirror direction [3,12]. Incident solar radiation includes both direct sunlight and sunlight scattered by the atmosphere. If its band irradiance is $E_{k}^{g}$, a surface with reflectance $\rho_{k}$ reflects a flux per unit area of $\rho_{k}E_{k}^{g}$. For a Lambertian surface, reflected radiance is independent of outgoing direction. Under the horizontal-surface approximation used here, it is given by [12]

$$L_{k}^{\mathrm{ref},\mathrm{sol}}=\rho_{k}E_{k}^{g}/\pi ,\;\rho_{k}=1-\varepsilon_{k}\;(\mathrm{opaque}\;\mathrm{Lambertian}\;\mathrm{surface}).$$

The factor 1/π ensures that the total reflected flux over the outgoing hemisphere equals $\rho_{k}E_{k}^{g}$ per unit surface area. Sunlight scattered before reaching the surface is included in this solar term, not in the reflected thermal sky term.

Specular reflection depends on Fresnel reflectance and the relative directions of the sun, surface normal, and sensor. A bright glint occurs when the mirror direction aligns with the viewing direction. Under uniform averaging within a pixel, a smaller projected area fraction of the specular region gives a proportionally smaller contribution to the pixel-averaged radiance. Atmospheric attenuation acts along both the sun-to-surface and surface-to-sensor paths [12]. The solar inputs are calculated in Section 3.3; the reflecting area and alignment for the arched polyethylene (PE) roof are modeled in Section 3.5.

Taken together, these components contribute differently across spectral bands. Their differing spectral responses provide a basis for cross-band evaluation of the component predictions, beyond agreement in total radiance alone.

## 3. Methods

Figure 2 summarizes the application of the observation model (Equation (1)) to field imagery. Radiometric calibration, image-based geometry, and atmospheric calculations supply the inputs for sky-radiance prediction, ground-target decomposition, and surface-temperature retrieval.

### 3.1. Radiometric Calibration

Radiometric calibration establishes the relationship needed to convert the camera’s digital numbers (DN) into band radiance. A blackbody at known temperatures is imaged, and its radiance is calculated from the Planck function weighted by the sensor spectral response. The measured DN and calculated radiance are then paired to estimate gain and offset:

(3) $$DN=G\;L_{\mathrm{band}}\left(T\right)+O,\;L_{\mathrm{band}}\left(T\right)=\int B_{\lambda }\left(T\right)\;S\left(\lambda \right)\;d\lambda ,$$

where $B_{\lambda }\left(T\right)$ is Planck radiance, S(λ) is sensor spectral response, *G* is gain, and *O* is the radiation-independent offset. Measurements are taken after stabilization with an area blackbody close to the camera aperture. The BLUE and RED channels are operated in different sensitivity modes selected for their band-dependent incident radiances and response characteristics. Calibration coefficients are fitted to blackbody measurements acquired in each operating mode.

The cooled MWIR camera operates at different integration times, so both the radiative signal and the time-dependent dark contribution must be accounted for. Measurements across blackbody temperatures and integration times *t* are fitted jointly to separate these contributions [15]:

(4) $$DN=C\;t\;L_{\mathrm{band}}\left(T\right)+C_{DC}\;t+C_{0},$$

Here, *C* is the radiometric response coefficient, $C_{DC}$ is dark charge per unit integration time, and $C_{0}$ is fixed readout bias, giving G=Ct and $O=C_{DC}t+C_{0}$. For the fixed-integration-time LWIR detector, the fitted model is $DN=C^{\prime }L+b$, with $G=C^{\prime }$ and O=b [16,17]. Coefficients are estimated by least squares against band radiance [18].

To apply the calibration to field images, the radiative response and the additive frame correction are handled separately. Denote the response excluding the calibration offset $O_{\mathrm{cal}}$ by $R_{k}\left(L,t\right)$. The blackbody fit supplies the nominal linear MWIR response $C_{k}tL$. An empirical two-slope response correction is applied to low-radiance BLUE field data. The response is continuous at the transition, with a lower slope below it. Its common transition and slope settings are selected from sky-profile comparisons and specified in Section 4. An additive frame correction $D_{k}$ gives the field offset $O_{k}=O_{\mathrm{cal},k}+D_{k}$ and is determined as described in Section 3.4. Radiance is recovered by subtracting this offset and inverting the corresponding response.

### 3.2. Camera Attitude and Target Geometry

The atmospheric and reflection calculations require viewing direction and target range rather than pixel coordinates alone. These quantities are recovered after acquisition by combining features in the recorded images with satellite imagery and a digital elevation model (DEM). The procedure first relates pixels to camera-relative directions, estimates camera attitude from scene references, and then determines target locations and ranges.

Image projection.

An image projection relates a pixel’s distance from the image center to its angle from the optical axis. The wide-angle MWIR camera uses an equidistant projection with a third-order radial correction [19,20]:

(5) $$r=f_{0}\theta \left(1+k_{1}\theta^{2}\right),$$

In Equation (5), θ is the angle from the optical axis, *r* is the image radius, $f_{0}$ is the angular scale, and $k_{1}$ is the distortion coefficient. The auxiliary LWIR camera uses a constant angular scale given by its field of view divided by the pixel count.

Attitude estimation.

Camera attitude is needed to express these camera-relative directions as scene azimuths and elevations. Mapped scene features provide reference directions from the camera, with camera elevation checked against the DEM [21]. The solar direction is calculated from location and clock-corrected recording time and used as an additional reference. Known directions $\left(az_{j},el_{j}\right)$ are matched to observed image coordinates $p_{j}^{\mathrm{obs}}$ by minimizing

(6) $$\hat{\Phi }=\arg\min_{\Phi }\sum_{j}w_{j}{∥p^{\mathrm{proj}}\left(az_{j},el_{j};\;\Phi \right)-p_{j}^{\mathrm{obs}}∥}^{2}.$$

where Φ contains attitude and projection parameters and $w_{j}$ weights each reference by its uncertainty. Roll is fixed at zero for the level mounting; common features share reference directions across frames. LWIR yaw and pitch are determined from two reference objects per frame using its fixed angular scale.

Viewing directions and ranges.

The fitted projection and attitude are inverted to assign (el,az) to pixels. These directions identify the sky-viewing angles and the rays used for terrain-based target ranging. For terrain-based ranging, the viewing ray is intersected with the DEM [21]. In the local planar approximation, $z_{\mathrm{ray}}\left(s\right)=H+s\tan\left(el\right)$, where *H* is camera elevation and *s* is horizontal distance; the first terrain intersection locates the target. For targets identified in georeferenced satellite imagery, ranges are calculated directly from their mapped positions relative to the camera.

### 3.3. Atmospheric Radiative-Transfer Calculations

To separate surface and atmospheric contributions, the model requires target-path transmittance, atmospheric radiance along the viewing direction, and solar irradiance incident on the surface. These quantities are calculated using atmospheric inputs for the observations and the recovered geometry. The Line-By-Line Radiative Transfer Model (LBLRTM) supplies molecular target-path transmittance [22]; libRadtran with the discrete ordinate radiative transfer (DISORT) solver supplies thermal and scattered sky radiance and surface irradiance [23,24,25].

Atmospheric inputs.

Temperature, water vapor, and aerosol inputs are assembled to represent the atmospheric conditions during imaging. Because the low-elevation viewing paths traverse extended distances through the lower atmosphere, temperature and water vapor in this region are constrained using nearby surface air temperature and dew point at the recording times. Radiosonde profiles supply the background vertical structure, with the local adjustment tapered with height. Adjacent soundings, where used, are interpolated to the recording time [4]:

(7) $$X\left(z,t\right)=w\;X_{1}\left(z\right)+\left(1-w\right)\;X_{2}\left(z\right),\;w=\frac{t_{2}-t}{t_{2}-t_{1}},$$

The profiles $X_{1}$ and $X_{2}$ correspond to $t_{1}$ and $t_{2}$, respectively. Interpolation is linear for temperature and logarithmic for pressure and water vapor. The background profiles are adjusted in the lower atmosphere, with surface observations providing the local reference conditions; a standard atmosphere extends them above the measured height range. Data sources are listed in Section 4.

A standard continental aerosol spectrum [26] is scaled to observed visibility using the Koschmieder relation [27], following the visibility parameterization of MODerate resolution atmospheric TRANsmission (MODTRAN)-class models [28]. When visibility reaches the reporting ceiling, hourly particulate-matter concentration PM scales the extinction:

(8) $$k_{\mathrm{aer}}\left(\lambda ;\;t\right)=k_{\mathrm{aer}}^{\mathrm{ref}}\left(\lambda \right)\cdot \frac{PM(t)}{PM^{\mathrm{ref}}},$$

The reference extinction spectrum and particulate-matter concentration are denoted by $k_{\mathrm{aer}}^{\mathrm{ref}}$ and $PM^{\mathrm{ref}}$, respectively. This scaling changes extinction magnitude while holding aerosol spectral shape fixed. CO and O_3_ concentrations are updated from same-day air-quality observations.

Target-path transmittance.

Target-path transmittance determines how much surface radiation reaches the camera at each range. Ground-target paths are represented by homogeneous horizontal paths at the local atmospheric conditions, using the adopted target ranges. LBLRTM computes reference-path monochromatic optical depth; division by reference distance gives the attenuation coefficient σ(λ). The molecular band transmittance at each target range is

(9) $$\tau_{k}\left(R\right)=\frac{\int S_{k}\left(\lambda \right)\;e^{-\sigma (\lambda )\;R}\;d\lambda }{\int S_{k}\left(\lambda \right)\;d\lambda }.$$

Equation (9) is evaluated separately at each range to retain the different attenuation rates within the band. Aerosol attenuation is applied separately to the target predictions.

Sky radiance and surface irradiance.

Thermal emission and scattered sunlight are calculated separately so that their contributions can be compared in the sky images. The same solar calculation also supplies the illumination for surface-reflection predictions. libRadtran’s uvspec program uses DISORT with a plane-parallel atmosphere and correlated-*k* molecular absorption [23,24,25]. Separate thermal and solar runs use the same profile and aerosol inputs. With the solar source disabled, the thermal run gives sky radiance on the viewing-angle grid. The solar run uses the recording-time solar position to calculate scattered radiance and direct plus diffuse surface irradiance. Thermal runs use a coarser wavelength grid; solar runs use a finer grid to resolve the CO_2_ feature in surface irradiance.

Solar input adjustment.

The ratio of station-measured mean global irradiance to the corresponding clear-sky calculation over 0.3–2.8 μm is applied to the band surface irradiance $E_{k}^{g}$. This approximation applies the same relative irradiance reduction in the broadband and MWIR intervals. Its effect on the BLUE component prediction is evaluated using cloud scenarios in Supplementary Materials, Section S5.

Diffuse reflection uses irradiance integrated over incident directions, whereas the glint model represents a directional reflection of the solar disc. The latter therefore uses solar-disc radiance $L_{⊙,k}$, obtained by band-integrating Planck radiance at $T_{\mathrm{sun}}\approx 5800$ K. Sun-to-roof transmittance and the reflecting area selected by the finite solar disc are applied in Equation (15).

### 3.4. Per-Band Prediction of Sky Background Radiance

Sky regions provide a comparison of atmospheric radiance without a ground-surface contribution. The thermal and solar-scattering calculations are evaluated at each frame’s viewing angles and solar position, and an instrumental glare term is added to predict the signal recorded by the camera:

(10) $$L_{k}^{\mathrm{sky}}\left(el,\;\Delta \phi \right)=L_{k}^{\mathrm{path}}\left(el\right)+L_{k}^{\mathrm{scat}}\left(el,\;\Delta \phi \right)+L_{k}^{\mathrm{glare}}\left(\psi \right),$$

where $L_{k}^{\mathrm{path}}$ and $L_{k}^{\mathrm{scat}}$ are the thermal and solar-scattering outputs of Section 3.3. The glare term represents the additional sun-dependent instrument response separately from atmospheric scattering. Its decrease with angular distance ψ from the sun is represented by a power law:

(11) $$L_{k}^{\mathrm{glare}}\left(\psi \right)=A_{k}\;\psi^{-n},\;\cos\psi =\sin\left(el\right)\sin\left(el_{s}\right)+\cos\left(el\right)\cos\left(el_{s}\right)\cos\left(\Delta \phi \right),$$

This empirical power law represents additional instrumental glare as a function of the sun-view separation angle ψ, expressed in degrees. Over the observed angular range, the power-law decay provides a simple description of the signal decreasing away from the sun, motivated by power-law tails in optical scattered-light distributions [29]. The amplitude *A* has units of W·m^−2^·sr^−1^·degree^*n*^, and the dimensionless exponent *n* controls the angular decrease. The glare coefficients *A* and *n* were determined using only BLUE sky observations at multiple times. RED sky and ground-target observations were not used to select these coefficients, which were held fixed across dates and frames. For the sky comparison, $L_{\mathrm{RED}}^{\mathrm{glare}}=0.35L_{\mathrm{BLUE}}^{\mathrm{glare}}$.

Frame-offset correction.

Before comparing the angular profiles, an additive correction aligns the measured and predicted DN at one sky reference in each frame and band. The lowest valid elevation in the selected clear-sky region is used as this reference. At low elevations, the strongly absorbing RED channel is dominated by the lower atmosphere, providing the thermal reference [30,31]. Using the response defined in Section 3.1, $DN_{k}^{\mathrm{pred}}=R_{k}\left(L_{k}^{\mathrm{pred}},t\right)+O_{\mathrm{cal},k}$, the frame correction is

(12) $$D_{k}=DN_{k}^{\mathrm{meas}}\left(el_{\mathrm{ref}}\right)-DN_{k}^{\mathrm{pred}}\left(el_{\mathrm{ref}}\right),$$

The same $D_{k}$ is applied across the frame. Measured radiance is obtained by subtracting $O_{\mathrm{cal},k}+D_{k}$ and inverting the same response. The remaining elevations then assess the angular variation of the prediction. RMS is calculated at these elevations, excluding the reference; DN residuals use the forward response, including the BLUE low-radiance branch. Conversion for the forest and near-greenhouse comparisons uses the same lowest-valid-elevation rule, with the RED offset evaluated from the thermal-only sky prediction. Thus, the reference-selection procedure is shared, while the RED predictions used for the sky comparison and target conversion differ by the glare term. Offsets are interpolated in time when a target frame falls between sky references.

### 3.5. Radiance Decomposition and Surface Temperature Retrieval for Ground Targets

The forest and near-greenhouse analysis separates surface emission, reflected radiation, and atmospheric path emission in the measured signal. Surface temperature is first retrieved from RED, and the resulting component model is then evaluated against BLUE. The mean DN in each target region of interest (ROI) is converted to radiance using the response and field offset described above. For an opaque Lambertian surface, $t_{o,k}=0$ and $\rho_{k}=1-\varepsilon_{k}$ in Equation (1). With the isothermal-path approximation from Section 2.1.1, the four-term ground-target model is

(13) $$L_{k}=\tau_{k}\left(R\right)[\underset{\left(1\right)\;\mathrm{surface}\;\mathrm{emission}}{\underset{︸}{\varepsilon_{k}B_{k}\left(T_{s}\right)}}+\underset{\left(2\right)\;\mathrm{reflected}\;\mathrm{sky}}{\underset{︸}{\left(1-\varepsilon_{k}\right)\;L_{k}^{\mathrm{sky}↓}}}+\underset{\left(3\right)\;\mathrm{solar}\;\mathrm{reflection}\;\left(\mathrm{diffuse}\right)}{\underset{︸}{\left(1-\varepsilon_{k}\right)\;\frac{E_{k}^{g}}{\pi }}}]+\underset{\left(4\right)\;\mathrm{path}\;\mathrm{emission}}{\underset{︸}{\left(1-\tau_{k}\left(R\right)\right)\;B_{k}\left(T_{\mathrm{path}}\right)}}.$$

Emissivity is specified for each target and held fixed in the inversion. Forest values are band integrals of library spectra; the near greenhouse uses an adopted effective value, as specified with the target results. In the ground-target calculation, reflected thermal sky radiation uses $L_{k}^{\mathrm{sky}↓}\approx \varepsilon_{\mathrm{sky}}B_{k}\left(T_{\mathrm{air}}\right)$, with a fixed effective sky emissivity specified in Section 4. This equivalent radiance represents the thermal sky input for diffuse reflection; incident direct and scattered sunlight are included in $E_{k}^{g}$. The solar term uses cloud-adjusted horizontal-surface irradiance $E_{k}^{g}$; effective path temperature is set to local low-level air temperature, $T_{\mathrm{path}}=T_{\mathrm{air}}$.

Thermal retrieval and cross-band prediction.

RED has a smaller solar contribution than BLUE because of the solar spectrum and atmospheric absorption. Surface temperature is therefore retrieved using thermal terms (1), (2), and (4). The effects of omitting RED solar reflection on the retrieved temperature and BLUE prediction under the forest observation conditions are evaluated in Supplementary Materials, Section S3. With emissivity and atmospheric inputs fixed, the modeled radiance increases monotonically with surface temperature, allowing the temperature matching the measured RED radiance to be found by bisection:

(14) $$L_{k}^{\mathrm{meas}}=\tau_{k}\left[\varepsilon_{k}B_{k}\left(T_{s}\right)+\left(1-\varepsilon_{k}\right)L_{k}^{\mathrm{sky}↓}\right]+\left(1-\tau_{k}\right)B_{k}\left(T_{\mathrm{path}}\right)\;\;\Rightarrow \;\;T_{s}^{\left(k\right)}.$$

The retrieved temperature is held fixed in the full BLUE prediction, including diffuse solar reflection, with no further target fitting. This comparison checks whether one surface temperature and the calculated component contributions account for both bands despite their different atmospheric attenuation and solar sensitivity. Measured BLUE excess over that prediction is examined for directional reflection. The same thermal inversion is applied independently to LWIR for the opaque forest target, providing a temperature comparison with a different detector and atmospheric window.

Specular reflection from arched roofs.

For the distant PE roofs, measured BLUE peaks are compared with a thermal and diffuse-reflection baseline and an idealized specular contribution to assess the radiance associated with localized brightening. This analysis retains band offsets obtained by averaging sky-reference residuals over the sampled elevations, using a thermal-only RED prediction. Their diffuse baseline uses $T_{s}=T_{\mathrm{air}}$, with an effective path temperature matched to the RED ROI radiance. The measured BLUE peak minus this baseline gives the excess to be examined. To assess whether solar reflection can account for its scale, the roof is approximated by a circular cylinder. The normal required for reflection bisects the roof-to-sun and roof-to-camera directions; cylinder normals are perpendicular to the ridge axis. A cross-ridge azimuth inferred from the first June event is used to compare the June and July geometries.

An ideally aligned patch provides a radiance reference, calculated separately for each June solar position. The finite solar disc selects a narrow arc of reflecting surface. Its projected area within a pixel gives

(15) $$\Delta L_{k}^{\mathrm{ref}}\approx \rho_{F}L_{⊙,k}\;\tau_{\mathrm{sun},k}\;\tau_{k}^{\mathrm{sol}}\left(R\right)\;\frac{A_{\mathrm{glint},\mathrm{proj}}}{A_{\mathrm{pix}}}.$$

In this expression, $\rho_{F}$ is the unpolarized Fresnel reflectance, $L_{⊙,k}$ is the solar-disc band radiance, and $\tau_{\mathrm{sun},k}$ and $\tau_{k}^{\mathrm{sol}}\left(R\right)$ are sun-to-roof and roof-to-sensor transmittances weighted for the solar source. $A_{\mathrm{glint},\mathrm{proj}}$ is the reflecting area projected perpendicular to the viewing direction, and $A_{\mathrm{pix}}$ is the pixel area in that same plane. The area is evaluated by vector reflection over the solar disc and integration of projected surface elements, using parallel rays and a square footprint centered on the strip with one edge along the projected ridge. This accounts for both the selected arc width and foreshortening along the ridge.

The reference adopts radius r=2.75 m, solar diameter 0.534°, $L_{⊙,k}=1.11\times 10^{5}$ W m^−2^ sr^−1^, $\tau_{k}^{\mathrm{sol}}\left(5.5\;\mathrm{km}\right)=0.58$ including aerosols, and $\rho_{F}\approx 0.075$ at incidence angle 55.4–55.5°. The nominal IFOV gives a pixel side of 21.1 m and $A_{\mathrm{pix}}=446$ m^2^; the calculated reflecting area is approximately 0.22–0.23 m^2^. This idealized geometry provides a reference scale for interpreting the measured excess radiance.

## 4. Experiments

This section describes the observations and experimental conditions used to evaluate the radiance-decomposition method, including the instruments, site and dates, analysis targets, and calibration and meteorological data.

### 4.1. Instruments

The observations use a primary dual-band MWIR camera and an auxiliary LWIR camera (Table 1). The wavelength ranges used in the analysis are 3.5–4.2 μm for the shorter band, BLUE, and 4.4–5.2 μm for the longer band, RED. The two bands lie on opposite sides of the CO_2_ absorption feature near 4.3 μm. The decomposition uses the differences in atmospheric transmittance and solar contribution between these bands. The spectral-response approximation used for band integration, reference-temperature band-integrated blackbody radiances, solar-disc radiances, and target-path transmittances are provided in Supplementary Materials, Section S1.

The camera uses a 512×512 cooled photon detector. A beamsplitter gives the two bands a common field of view, allowing simultaneous comparison of the same scene. Its variable integration time enables separation of the dark-current term during calibration (Section 3.1). Image-based geometry estimation gives an effective horizontal field of view of 100.3° (Section 5.1) and a nominal IFOV of 3.84 mrad per pixel. This wide view represents persistent area surveillance, in which distant targets occupy only a few pixels or less (Section 1).

The auxiliary camera is an uncooled 640×480 LWIR microbolometer with an 8–14 μm passband, operated at a fixed 30 Hz without varying integration time. Its horizontal field of view is 22.8°, roughly one quarter of the primary camera’s, and its IFOV is 0.62 mrad per pixel, approximately six times finer. The LWIR additive correction was obtained by averaging the corrections from six sky strips at elevations of 1°, 1.5°, 2°, 3°, 4°, and 5° within the images. LWIR observations were used for the ground-target temperature comparison. This camera provides an independent thermal retrieval for ground targets within its field of view (Section 5.3.1).

### 4.2. Site and Dates

Observations were acquired from the rooftop of a 16 m building in Yongin, Republic of Korea (37.14398° N, 127.17945° E), at an observation altitude of 146 m above sea level (ASL) with an open view to the south–west. In this study, low-altitude observations refer to the radiative paths from this ground-based camera toward near-surface targets and the sky at low elevation angles. Recording took place on the afternoon of 11 June 2026 and the morning of 1 July 2026. The June observations comprised two sessions around 15:00; the July ground-target observations comprised four sessions between 09:54 and 11:57. Paired LWIR observations are available for the first three July sessions.

Table 2 lists the conditions for these ground-target sessions. June was clear, dry, and highly visible. July began humid and overcast and cleared during the morning. This contrast provides a test of model consistency between two atmospheric conditions.

### 4.3. Targets

The radiance targets comprise vegetation in near and far forests and translucent polyethylene (PE) greenhouses capable of specular reflection. These targets span distances from 280 m to 5.5 km.

Radiance decomposition and temperature retrieval are performed for the near and far forests and the near greenhouse. The two distant greenhouse buildings are examined separately for specular reflection. Sky radiance is sampled across elevation within a clear image region. The narrow-field LWIR observations cover a subset of the ground targets.

The sky comparison uses four July frames at 09:33, 10:56, 11:23, and 11:57 Korea Standard Time (KST) and two June frames at 14:55 and 15:01 KST, after correction of the instrument clock. Samples are the median DN within the selected region and ±1.2° of the nominal elevations 6,10,15,22, and 30°. The lowest valid elevation is 6° in five frames and 10° in the June 14:55 frame, whose selected region does not include the 6° bin. After excluding the offset reference, four elevations per frame are compared, or three for the June 14:55 frame.

### 4.4. Calibration and Reference Measurements

To establish the DN–radiance relation, MWIR calibration uses a blackbody sweep over eight temperatures and six integration times. LWIR calibration uses eight temperatures from 15 to 50 °C. A high-emissivity area blackbody is placed close to the camera aperture so that intervening atmospheric absorption is negligible.

Field processing uses a continuous BLUE response with a low-radiance slope of 0.7Ct below the band radiance of a 12 °C blackbody, joining the nominal CtL response at that transition. The glare coefficients were selected by a grid search over *A* and *n* using BLUE sky observations. For each candidate, a mean DN offset was estimated within each frame, and the sum of squared brightness-temperature residuals was minimized over the valid elevations at the seven times used for coefficient selection. The selected values, $A_{\mathrm{BLUE}}=1000$ and n=2.5, are fixed across observations; the additive correction for the manuscript’s sky comparisons is determined at the lowest valid elevation for each band and frame. The incident thermal sky radiance used in the ground-target reflection term was approximated as $\varepsilon_{\mathrm{sky}}B_{k}\left(T_{\mathrm{air}}\right)$, informed by representations of downwelling long-wave radiation using air temperature and effective emissivity [32]. A common value of 0.85 was applied to both days; surface reflectance and target-path transmittance were applied to this sky input to obtain the reflected contribution at the camera. Its effects on temperature retrieval and BLUE prediction are evaluated in Supplementary Materials, Section S4.2.

Meteorological observations were obtained from the Korea Meteorological Administration (KMA). Surface air temperature, dew point, visibility, and cloud amount were obtained from the Icheon Automated Synoptic Observing System (ASOS) station (203), approximately 30 km from the imaging site, and solar irradiance from Suwon ASOS. Icheon air temperature and dew point constrained the lower-atmospheric conditions at the recording times. Radiosonde data from Changwon (station 47155), approximately 253 km from the imaging site, supplied the background vertical structure. Air-quality and particulate-matter data are from four AirKorea sites in Yongin.

## 5. Results

The results first establish the radiometric calibration and observation geometry, then compare predicted radiance components with sky and ground-target measurements in both MWIR bands. The final analysis examines localized brightening from PE roofs using the specular-reflection model.

### 5.1. Radiometric Calibration and Observation Geometry

Radiometric calibration.

Figure 3 shows blackbody calibration over 5–40 °C for MWIR and 15–50 °C for LWIR. After subtraction of dark current and fixed bias, the six MWIR integration-time datasets follow a common line against radiance × integration time, consistent with the response model in Section 3.1. These fits establish the blackbody-derived calibration response. LWIR follows a linear DN–radiance relation at fixed integration time.

The MWIR fit gives $R^{2}=0.99996$ over 48 measurements, with residual RMS values of 6.4 DN in BLUE and 13.0 DN in RED. The LWIR fit gives $R^{2}=0.99963$ over 8 measurements (Figure 3). Table 3 lists the adopted coefficients.

Observation geometry.

Figure 4 relates the image targets to satellite imagery and a DEM gridded from 1:5000 topographic contours. Together, these data provide the reference geometry for assigning viewing directions and ranges to the targets.

The fitted projection (Equation (5)) has $f_{0}=260.5$ px/rad, $k_{1}=0.157$, and an effective field of view of 100.3°. Figure 5 shows the resulting elevation contours and target reprojections, with a target reprojection RMS of 1.8 px.

Table 4 gives the resulting viewing angles and adopted ranges. The listed scene features are viewed downward, with el=−1.8 to −13.2°. The geometry references and radiance targets span distances from 280 m to 5.5 km. The unobstructed south-westerly corridor in Figure 4d is consistent with visibility of this target.

### 5.2. Sky Background: Component Comparison and Atmospheric Sensitivity

The sky comparisons evaluate the modeled thermal and solar-related contributions across observation times and viewing directions. Common sky-model parameters are selected from the observations and then held fixed across frames. We first compare atmospheric transmittance in the BLUE and RED bands along the paths from the ground targets to the camera.

Atmospheric band contrast.

Using atmospheric inputs adjusted with surface observations for 1 July and 11 June, LBLRTM calculates molecular transmittance along the horizontal target paths described in Section 3.3. The two date-specific calculations give RED transmittances of 0.39–0.47 at 842 m and 0.08–0.13 at 5.5 km, whereas BLUE retains 0.60–0.67 at 5.5 km; each range spans the two atmospheric inputs. These values exclude aerosol attenuation, which is included separately in the target predictions (Section 3.3). This contrast underlies the increasing RED path-radiance fraction in the ground-target decomposition and the greater transmission of solar-reflected signals in BLUE. The sky comparison below examines the associated thermal and solar contributions along upward viewing paths.

Angular behavior and radiance components.

Figure 6 compares the observed sky radiance with the predicted total radiance and its thermal, scattering, and glare components at six observation times. Radiance generally decreases with viewing elevation as the atmospheric thermal contribution decreases. RED is dominated by thermal emission, whereas BLUE has a larger relative contribution from solar scattering and glare. The relative contributions vary between the July morning and June afternoon observations, allowing the common model to represent distinct angular profiles under different atmospheric conditions and solar-viewing geometries. After correction at the lowest valid elevation, RMS over the remaining measurements is 0.0072–0.0202 W m^−2^ sr^−1^ in BLUE and 0.0027–0.0274 W m^−2^ sr^−1^ in RED. In camera digital numbers (DN), the corresponding RMS errors are 13.5–24.5 in BLUE and 7.5–76.6 in RED.

The distinction is most visible in the June afternoon frames. At 30°, the modeled BLUE glare contribution is 0.060–0.065 W m^−2^ sr^−1^, compared with 0.015–0.031 in July. It partially compensates for the decreasing thermal contribution, producing a flatter total-radiance curve at higher elevations. The RED glare term remains below 2% of the modeled total in all six frames, preserving the contrast between a predominantly thermal RED response and a more solar-sensitive BLUE response.

Comparison with a standard atmosphere.

Figure 7 examines the sensitivity to the thermal atmospheric profile using the July 11:23 and June 15:01 frames. The same-day profile is replaced by the Air Force Geophysics Laboratory (AFGL) mid-latitude summer atmosphere [28], while aerosol settings, scattering, and glare remain fixed. The offset obtained from the same-day 6° prediction is retained for every profile, and RMS is calculated at 10–30°. This isolates the effect of changing the thermal profile under a common radiometric correction.

The unmodified standard atmosphere underpredicts both bands. Its BLUE RMS is 0.037–0.050 W m^−2^ sr^−1^, compared with 0.007–0.012 for the same-day profiles. The difference is larger in RED. At 11:23 on 1 July, the RMS error between observed and predicted sky radiance is 0.29968 W m^−2^ sr^−1^ with the standard atmosphere and 0.02742 W m^−2^ sr^−1^ with the observation-constrained profile. The latter is approximately 1/10.93 of the former. At 15:01 on 11 June, the corresponding RMS errors are 0.25224 and 0.01638 W m^−2^ sr^−1^, giving a ratio of 15.40. The same offset is retained across profiles within each image. In camera pixel values, these RED RMS errors correspond to approximately 793–837 DN with the standard atmosphere and 51–77 DN with the same-day profiles for the same two frames.

Replacing only the standard profile’s lowest-level temperature reduces RED RMS to 0.130 in July and 0.106 W m^−2^ sr^−1^ in June. Additionally scaling its water vapor to the same-day surface value reduces the July residual to 0.071 but increases the June residual to 0.136 W m^−2^ sr^−1^. Surface constraints therefore reduce much of the mismatch, but do not reproduce the same-day angular curves in either case. This comparison supports the use of locally constrained atmospheric inputs without attributing all remaining differences to a single profile parameter.

### 5.3. Component Decomposition and Surface Temperature Retrieval for Ground Targets

For each target, the temperature retrieved from RED is held fixed in the BLUE prediction, with no additional target parameter fitted (Section 3.5). The comparisons test the four-term model in Equation (13) across natural and artificial surfaces.

#### 5.3.1. Forest: Dual-Band Decomposition and LWIR Cross-Check

The near and far forests provide a comparison of thermal components at different ranges, with auxiliary LWIR observations providing a separate temperature check for the far forest.

Forest emissivities of 0.946 in BLUE and 0.949 in RED were obtained from the median band values of 76 Quercus samples from the ECOsystem Spaceborne Thermal Radiometer Experiment on Space Station (ECOSTRESS) spectral library and applied at both ranges and all six observation times [33]. The effects of varying forest emissivity and aerosol optical depth on retrieved temperature, BLUE radiance components, and total predicted radiance are presented in Supplementary Materials, Section S4.1. For the near (280 m) and far (1080 m) forests, observed BLUE radiance was compared with BLUE predictions calculated using the RED-retrieved temperatures (Figure 8). Across the 12 comparisons at six observation times, the largest difference between observed and predicted radiance was 0.056 W m^−2^ sr^−1^ (maximum absolute residual), and the RMS error was 0.0329 W m^−2^ sr^−1^. Residuals are observed minus predicted radiance; the individual comparisons are given in Supplementary Materials, Section S2. RED path emission contributes an average of 38% and 65%, respectively, allowing the cross-band comparison to test both surface-dominated and path-dominated observations. The RED-retrieved temperatures of the near and far canopies agree within 0.7 °C in five frames; the final June frame has a 3.8 °C difference, when low far-target transmittance increases retrieval sensitivity.

Because RED determines the temperature, agreement in that band is built into the retrieval. LWIR target radiance was corrected using the mean sky offset described in Section 4.1 and inverted for temperature using the thermal-radiance equation with atmospheric transmission, path thermal emission, and reflected sky radiation along the 1080 m path. Surface emissivity was set to 0.9515, the median 8–14 μm band value from the ECOSTRESS data [33]. Independently retrieved LWIR temperatures provided a cross-check for the far forest and exceeded RED estimates by 0.7–2.6 °C in the three paired July observations (Figure 9).

#### 5.3.2. Greenhouse Film: Diffuse Solar Reflection

The near greenhouse is used to examine whether diffuse solar reflection accounts for the BLUE contribution beyond the thermal components.

For the near greenhouse at 842 m, a common effective emissivity of 0.90 was applied across observations, informed by the mid-infrared optical properties of PE [34] and allowing for possible film soiling and ageing. The effects of film-emissivity variation on solar reflection and BLUE residuals are presented in Supplementary Materials, Section S4.2. Diffuse solar reflection is the second-largest BLUE component and increases with cloud-adjusted irradiance (Figure 10). Between 09:54 and 11:57 in July, observed BLUE radiance increased from 0.556 to 0.798 W·m^−2^·sr^−1^, while the prediction using the RED-retrieved temperature at each time increased from 0.524 to 0.794 W·m^−2^·sr^−1^. This increase is accounted for by changes in surface thermal emission from 0.383 to 0.583 W·m^−2^·sr^−1^ and diffuse solar reflection from 0.054 to 0.113 W·m^−2^·sr^−1^. The maximum absolute residual across all six observations is 0.037 W·m^−2^·sr^−1^ under the baseline calculation conditions. The sensitivity assessment of wavelength-dependent cloud attenuation in Supplementary Materials, Section S5 changes the residual range, but predictions including diffuse solar reflection remain closer to the observations than those excluding it in scenarios using the reported cloud cover. These observations are away from the mirror condition, contrasting with the strong glint from the distant PE roofs examined in Section 5.4. The film is excluded from the LWIR temperature comparison because its high LWIR transmission admits radiation from the greenhouse interior.

### 5.4. Specular Solar Reflection from PE Film

The two PE greenhouse buildings at 5.5 km exhibit localized BLUE peaks well above the thermal and diffuse-reflection baseline. Figure 11 shows the June images six minutes apart on the same radiance scale: the left building fades while the right remains bright. The peak excess above the baseline decreases from 1.16 to 0.43 W m^−2^ sr^−1^ on the left and increases from 0.99 to 1.22 on the right. In July, the corresponding excesses are only 0.02–0.07 W m^−2^ sr^−1^.

We interpret the localized excess and the contrasting changes in the two buildings as solar glint. On a curved roof, the changing solar direction changes which surface regions reflect sunlight toward the camera. This provides a mechanism for the strong peaks and their different evolution over the six-minute interval.

The cylindrical-roof calculation tests whether a reflecting patch can supply radiance on the observed scale. With the cross-ridge azimuth of 355.5° inferred from the first June event, the modeled reflection directions are closer to the camera in June than in July. For the adopted radius of 2.75 m, Equation (15) gives an ideal-alignment contribution of 1.74–1.79 W m^−2^ sr^−1^, exceeding the four June peak excesses but comparable in scale to the three strongest peaks (Figure 12). This calculation checks whether the glint interpretation accounts for the magnitude of the observed excess under the assumed roof geometry.

## 6. Discussion

The experimental results show that joint analysis of the two MWIR bands helps assess the components contributing to the observed radiance. Agreement in total radiance in one band alone does not establish whether surface emission and atmospheric path emission have been appropriately apportioned. In this study, surface temperatures retrieved from RED are held fixed in the BLUE predictions, allowing the component model to be evaluated under a different spectral response. The comparisons for forests at different ranges and for the near greenhouse extend this evaluation across different atmospheric and surface contributions, while the forest LWIR comparison provides additional evidence for the thermal retrieval. The greenhouse sensitivity further shows that accounting for solar reflection improves the BLUE prediction across the tested cloud scenarios, although the residual magnitude depends on the illumination assumptions. Cross-band evaluation therefore offers a more specific test of the component interpretation than fitting each band’s total signal separately.

Taken together, the sky and ground-target analyses show that the contrast between the bands reflects differences in radiance composition, not simply signal magnitude. Atmospheric absorption in RED attenuates distant surface signals while increasing the relative contribution of thermal path emission. BLUE retains more information from distant surfaces but is also more sensitive to solar radiation. A bright BLUE feature therefore cannot be attributed to a high surface temperature alone, nor does a strong RED signal necessarily indicate strong surface emission. The localized excess radiance and temporal changes of the distant PE roofs illustrate the need for this distinction; their solar-reflection interpretation draws on spatial and temporal observations alongside the component predictions. These findings suggest that assessing the utility of a band for low-elevation infrared observations requires considering how range, background, and illumination affect the contributing components, rather than target signal magnitude alone.

Recovering the viewing geometry is also important for applying this component analysis to field imagery. Camera attitude and target ranges, which were not separately measured during acquisition, were estimated from image reference points, satellite imagery, and terrain data to establish the atmospheric paths corresponding to the observations. This demonstrates that field imagery without dedicated attitude and range measurements can still support quantitative radiance analysis when identifiable reference features and terrain data are available. Geometry recovery and cross-band comparison serve complementary roles: the former establishes the observation paths, and the latter evaluates the component predictions. The approach thus connects differences observed in dual-band field imagery to viewing geometry and radiance components, extending the analysis beyond a comparison of image brightness.

## 7. Conclusions

This study presented a method for decomposing atmospheric and surface contributions to low-elevation infrared radiance using dual-band MWIR observations and evaluating the component predictions across bands. For forests and the near greenhouse, surface temperatures retrieved from RED were held fixed in BLUE radiance predictions without further target fitting, linking the thermal retrieval in one band to the component prediction in the other. Agreement between far-forest temperatures and independent LWIR retrievals within 2.6 °C supported these temperature estimates. Sky observations provided an evaluation of the atmospheric contribution: observation-constrained atmospheric inputs reduced RED RMS error by a factor of approximately 11–15 relative to an unmodified standard atmosphere in two comparison frames under the same offset correction. These comparisons assessed ground-target surface-temperature retrievals and atmospheric radiance predictions for the sky against observations, providing evidence for the component interpretation.

Spatial and temporal observations were also used to interpret localized brightening that thermal components alone could not explain. For the distant PE roofs, BLUE radiance exceeding the thermal and diffuse-reflection predictions and the contrasting temporal changes of the two buildings were interpreted as solar glint, with an idealized reflection calculation used to examine its magnitude. By connecting band-specific component predictions to the characteristics of the observed scene, this approach provides a way to interpret infrared brightness differences in terms of atmospheric and solar contributions as well as surface temperature.

## Figures and Tables

**Figure 1:**
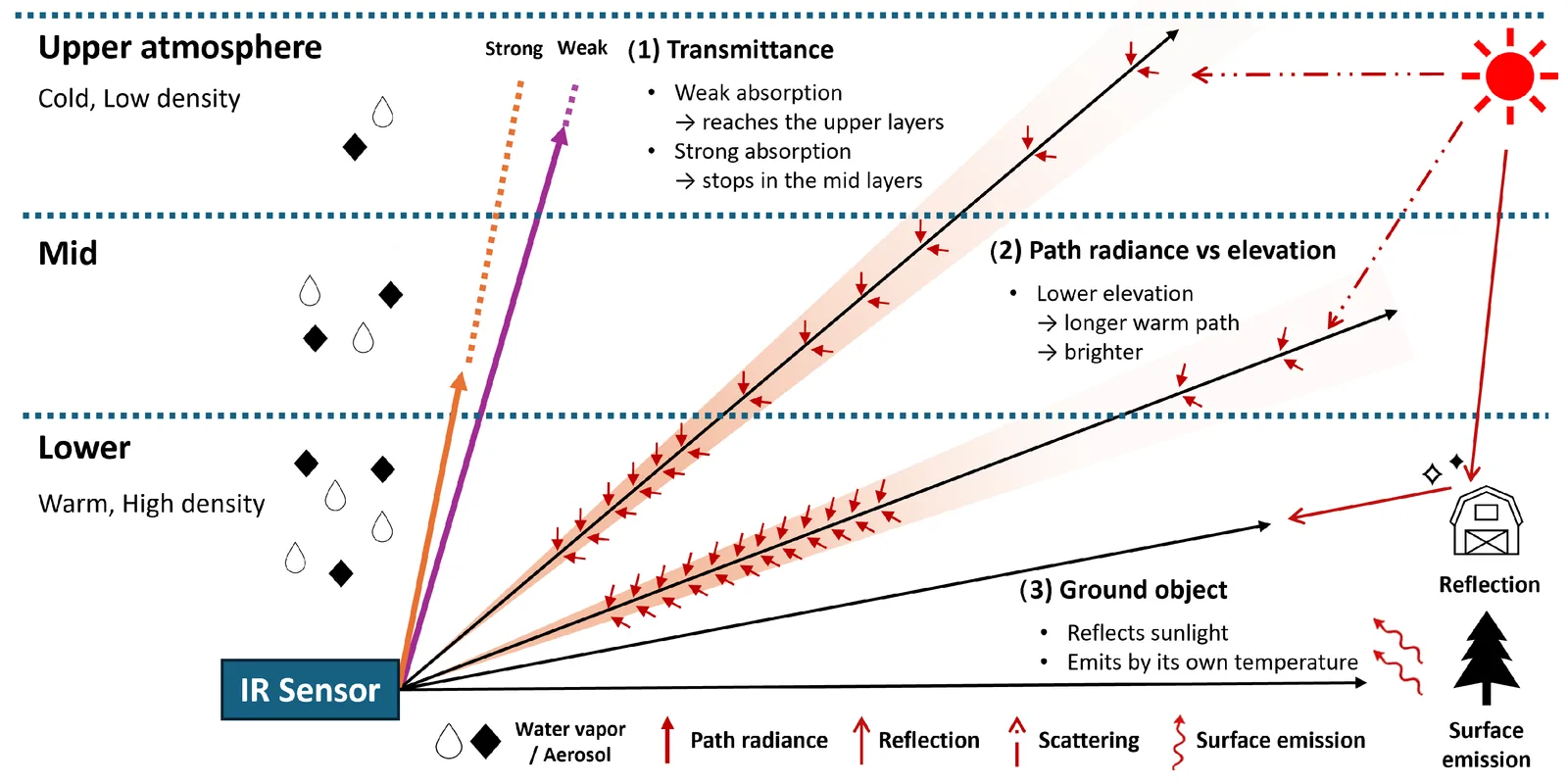
Conceptual decomposition of slant-path radiance. Lower, middle, and upper atmospheric layers illustrate (1) band-dependent penetration depth, (2) the elevation dependence of accumulated path radiance, and (3) ground-target emission and reflection. The lower layers are warmer and denser than the upper layers. Droplets represent gaseous water vapor, not cloud droplets; black diamonds represent aerosol. IR: infrared.

**Figure 2:**
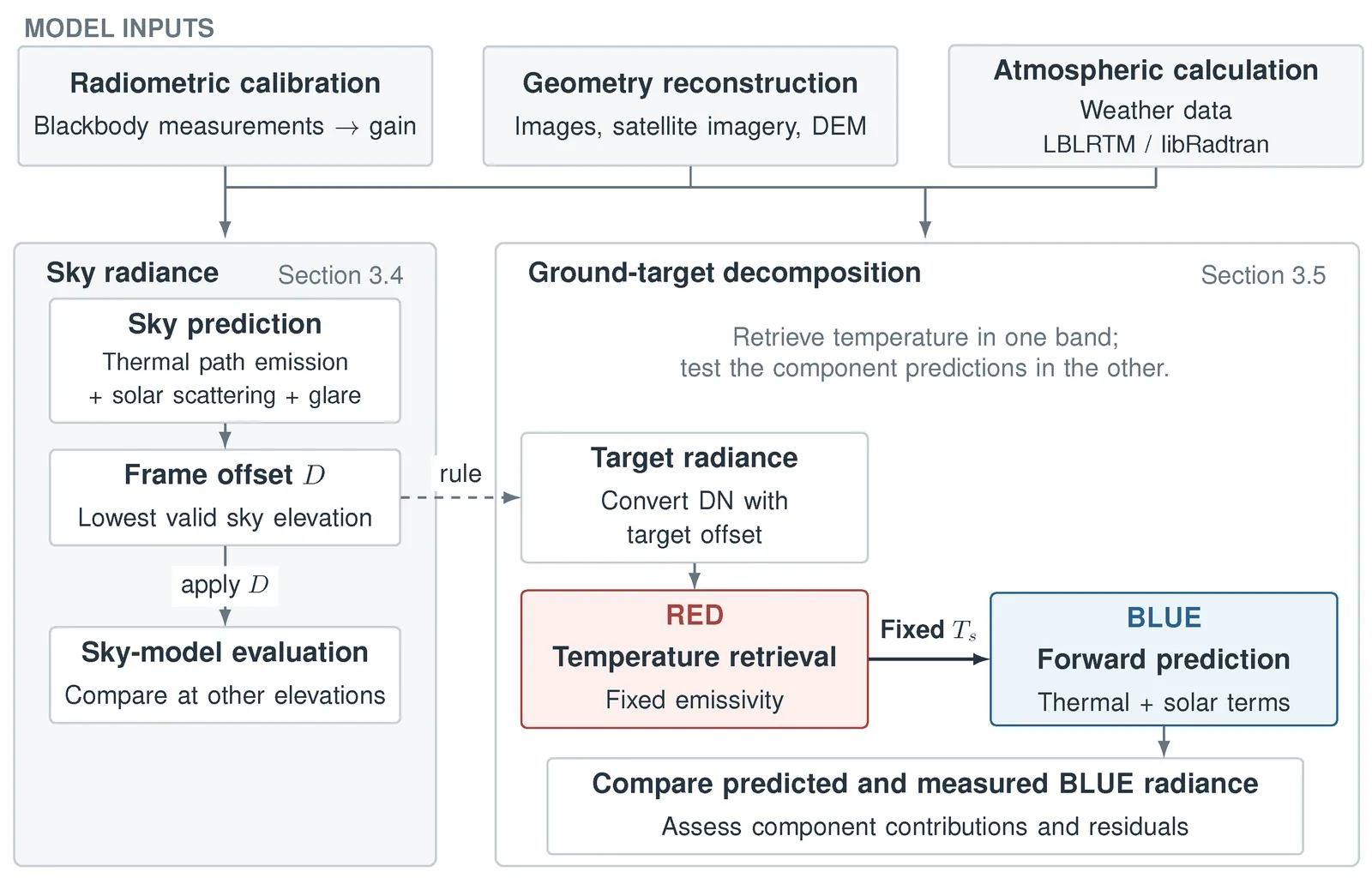
Model-based radiance decomposition for sky, forest, and near-greenhouse observations. The dashed arrow indicates the common sky-reference correction rule; target conversion evaluates its RED offset using the thermal-only sky prediction (Section 3.4). With emissivity specified for each target, RED determines surface temperature $T_{s}$, which is held fixed in the BLUE forward calculation. These BLUE comparisons introduce no additional fitted target parameter. The distant-roof reflection case is treated separately in Section 3.5. DN: digital numbers; DEM: digital elevation model; LBLRTM: Line-By-Line Radiative Transfer Model. BLUE and RED denote the shorter- and longer-wavelength bands, respectively.

**Figure 3:**
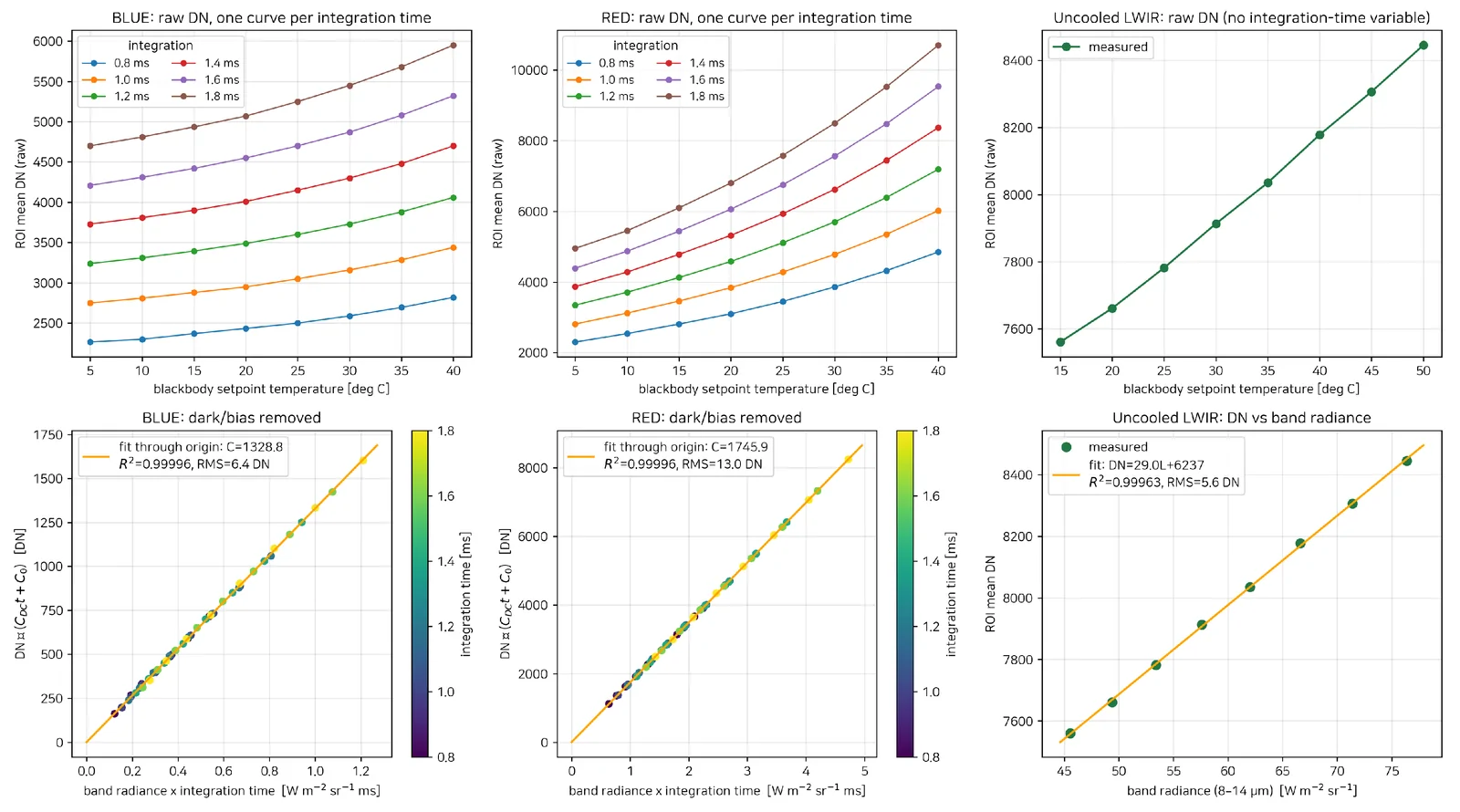
Radiometric calibration of the three detector channels. **Upper row:** Raw DN versus blackbody temperature, with MWIR curves shown for each integration time. **Lower row:** MWIR counts after subtraction of dark current and fixed bias versus radiance × integration time; LWIR raw DN versus radiance, with a linear fit including an offset. The six MWIR integration-time datasets follow a common line through the origin. MWIR colors identify integration time. RMS: root mean square.

**Figure 4:**
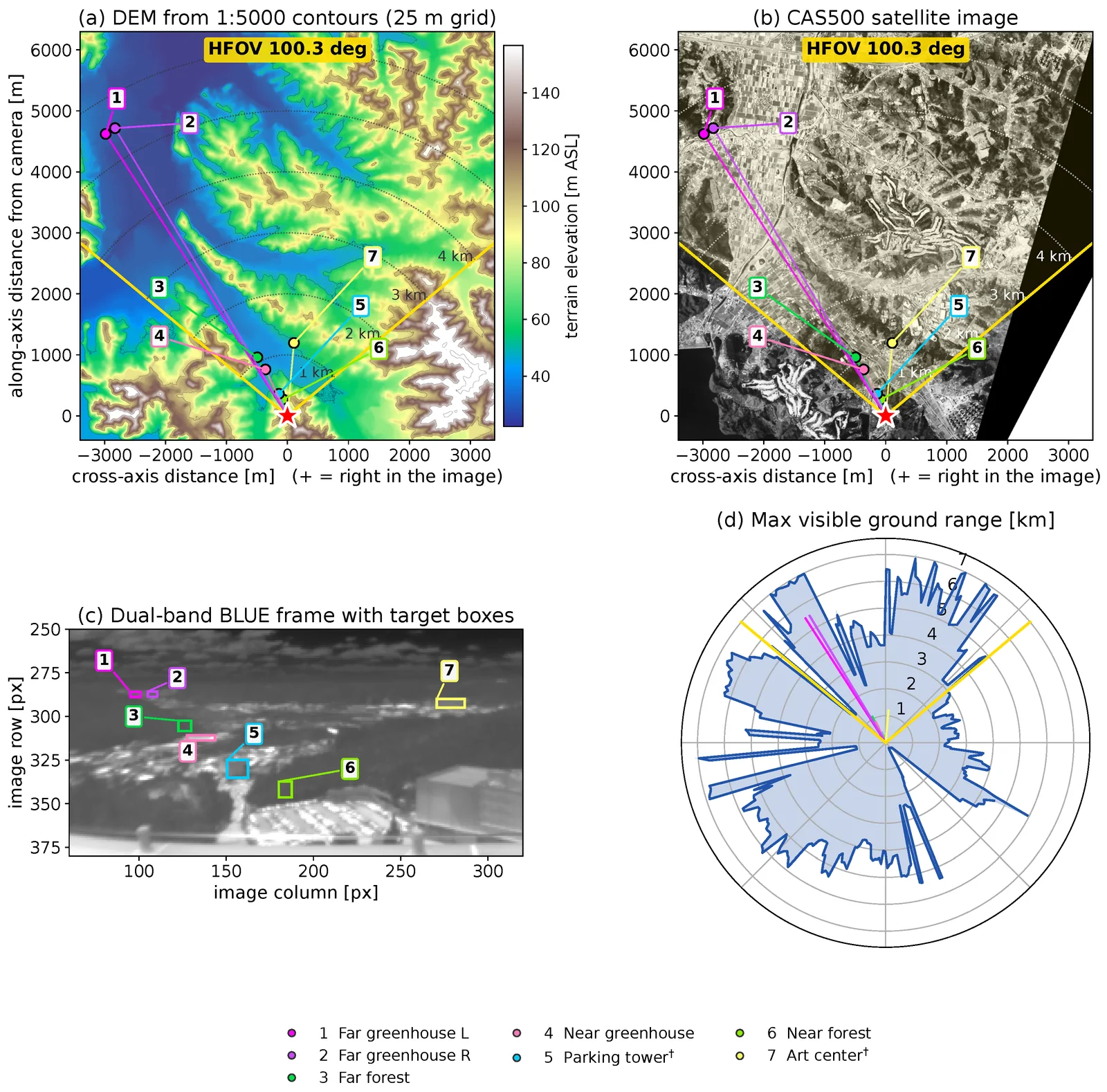
Reference geometry and image-to-ground correspondence. (**a**) DEM from 1:5000 topographic data; (**b**) Compact Advanced Satellite 500 (CAS500) imagery; (**c**) raw BLUE image with target boxes; (**d**) maximum visible ground range by azimuth. Numbered callouts identify the same scene features across panels; daggers in the legend denote features used only as geometry references. Panels (**a**,**b**,**d**) are oriented along the optical axis to match the left-to-right order in (**c**). The yellow sector denotes the effective field of view, and dotted arcs mark 1 km intervals. Satellite imagery: National Geographic Information Institute, Republic of Korea (CAS500 imagery, https://www.data.go.kr/data/15101976/fileData.do, accessed on 30 October 2026), used under Korea Open Government License Type 1; processed and annotated by the authors.

**Figure 5:**
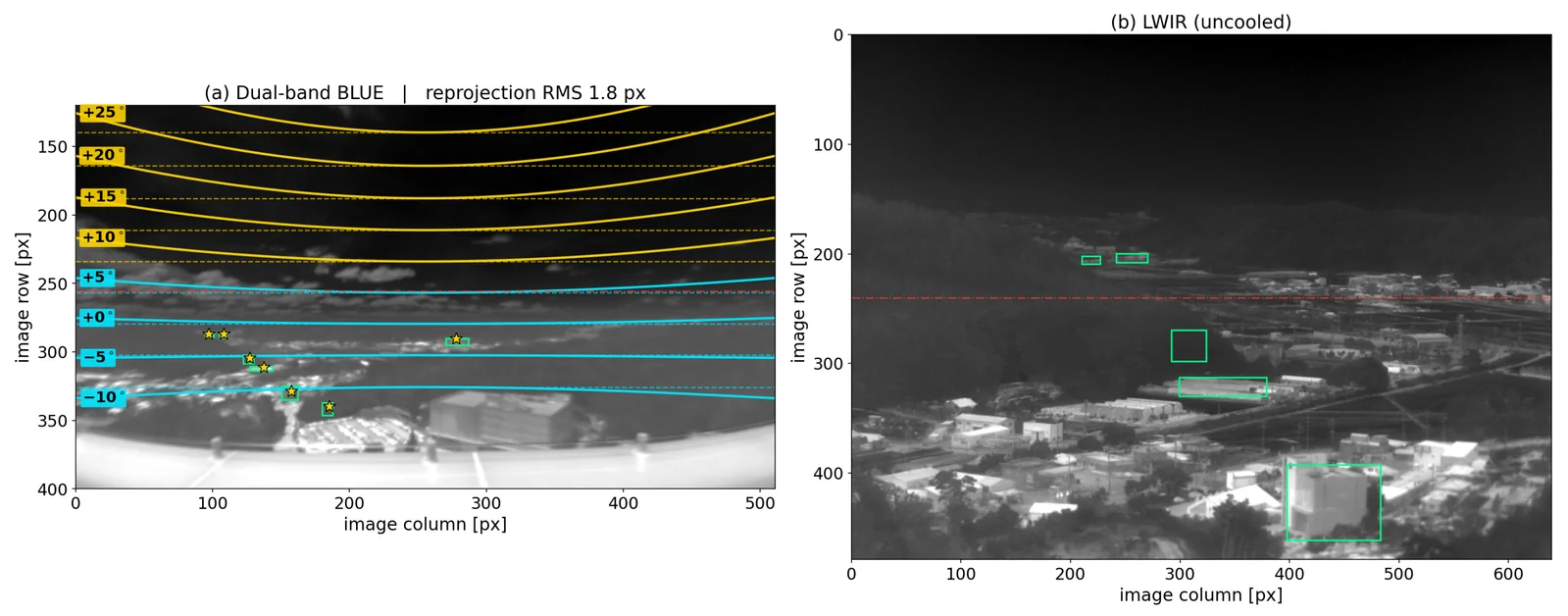
Constant -elevation contours and target reprojection. (**a**) BLUE image with elevation contours at 5° intervals (solid) and horizontal references through their central-column positions (dashed). Maximum departures from these references range from 2 to 46 px across the contours. Green boxes mark target annotations and yellow stars their reprojections, with RMS 1.8 px. (**b**) LWIR target annotations. Red dash-dotted lines mark the central image row in each panel.

**Figure 6:**
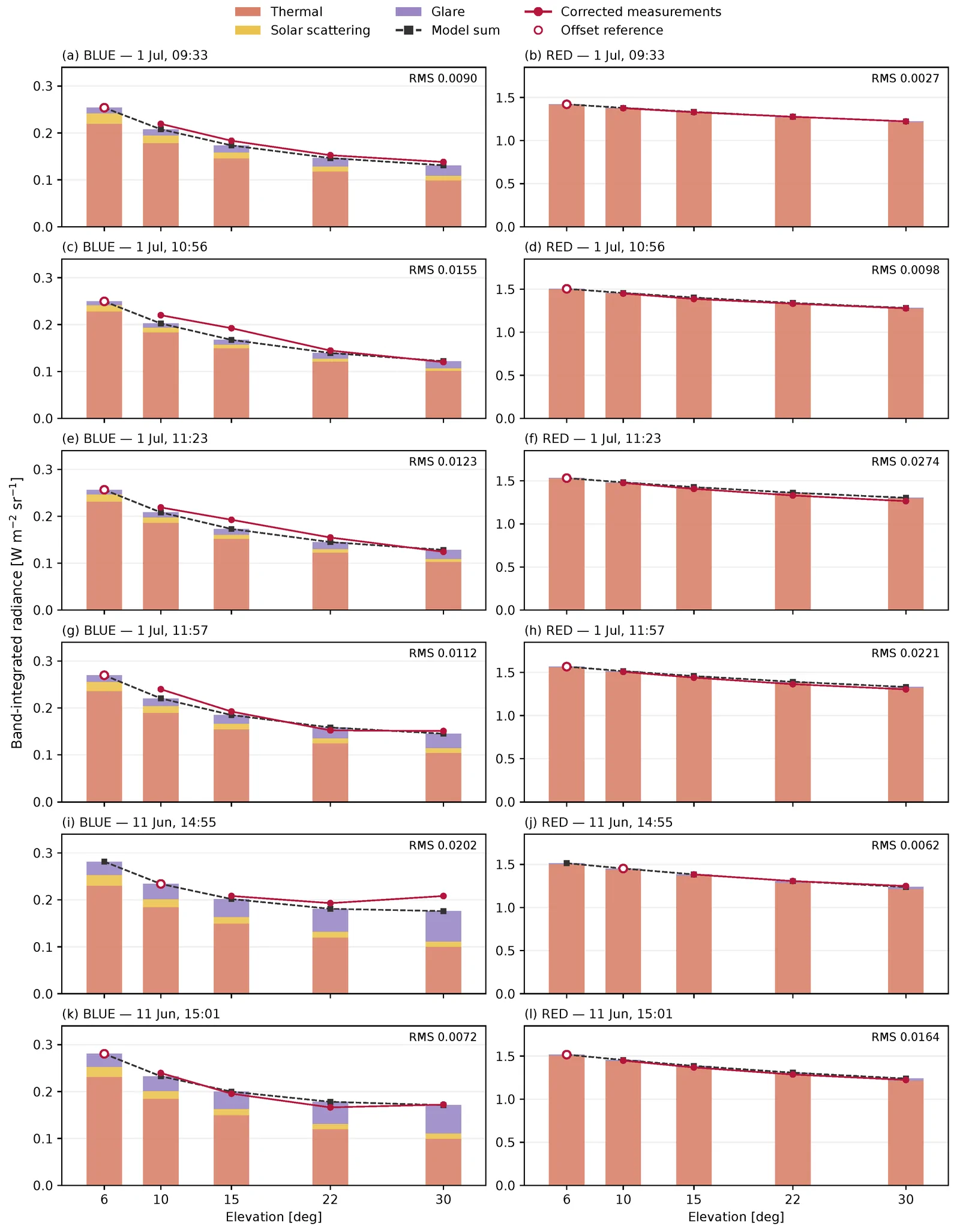
Sky-radiance components and corrected measurements for six frames. BLUE and RED are paired by recording time (KST). Bars show modeled thermal, scattering, and glare contributions; black dashed lines show their sum. Open circles identify the lowest valid elevation used for offset correction. Filled circles show the remaining measurements. Panel RMS values, in W m^−2^ sr^−1^, exclude the offset reference; the missing low-elevation measurement in the June 14:55 frame is not interpolated.

**Figure 7:**
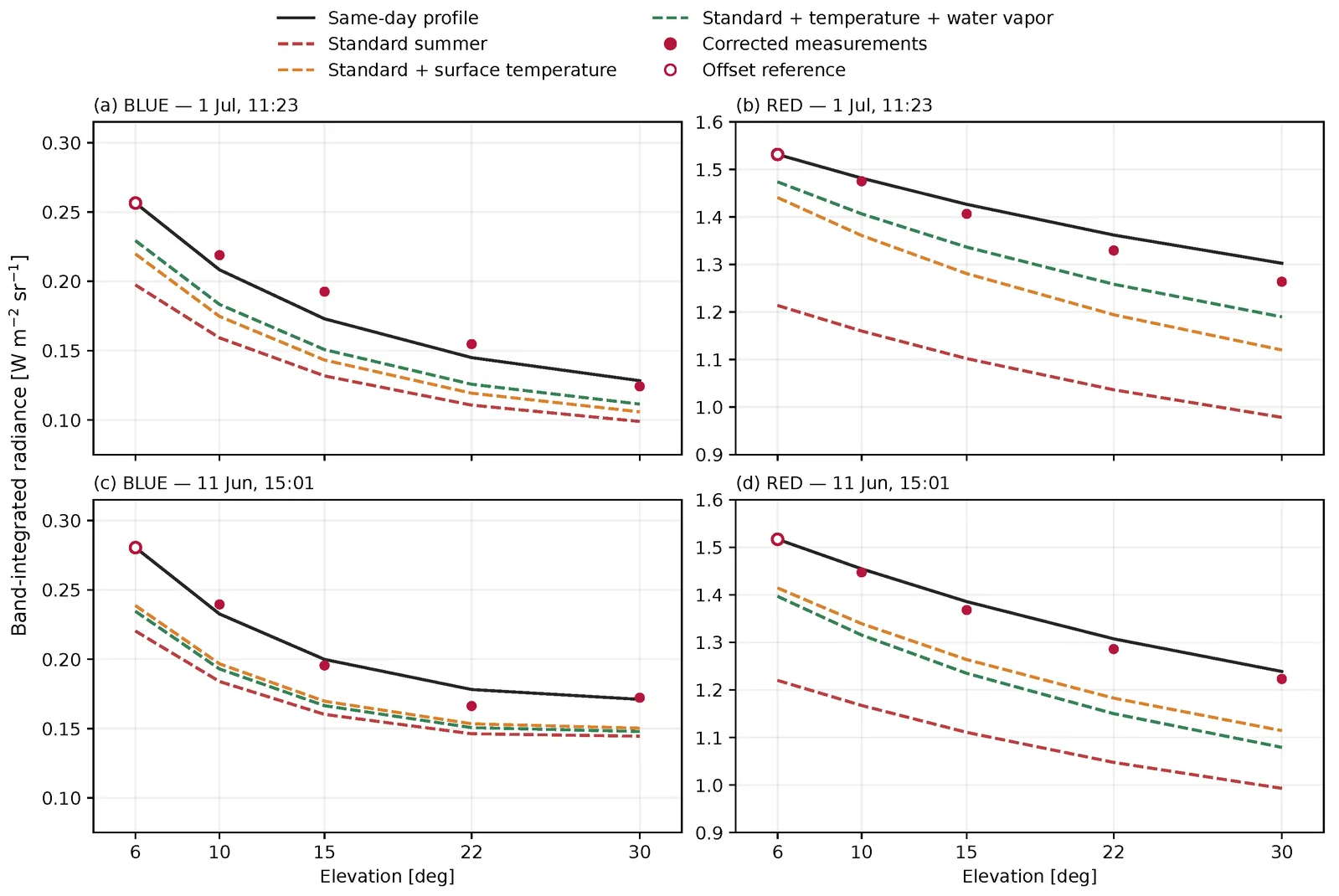
Sensitivity of sky radiance to the thermal atmospheric profile. Columns show BLUE and RED; rows show 1 July at 11:23 KST and 11 June at 15:01 KST. Black curves use the same-day profiles; dashed curves use the standard summer profile unchanged, with its lowest-level temperature replaced, or with that temperature replacement and water-vapor scaling to the same-day surface value. The same-day-derived frame offset, scattering, and glare are held fixed in all comparisons. Open circles mark the offset reference, which is excluded from the reported RMS.

**Figure 8:**
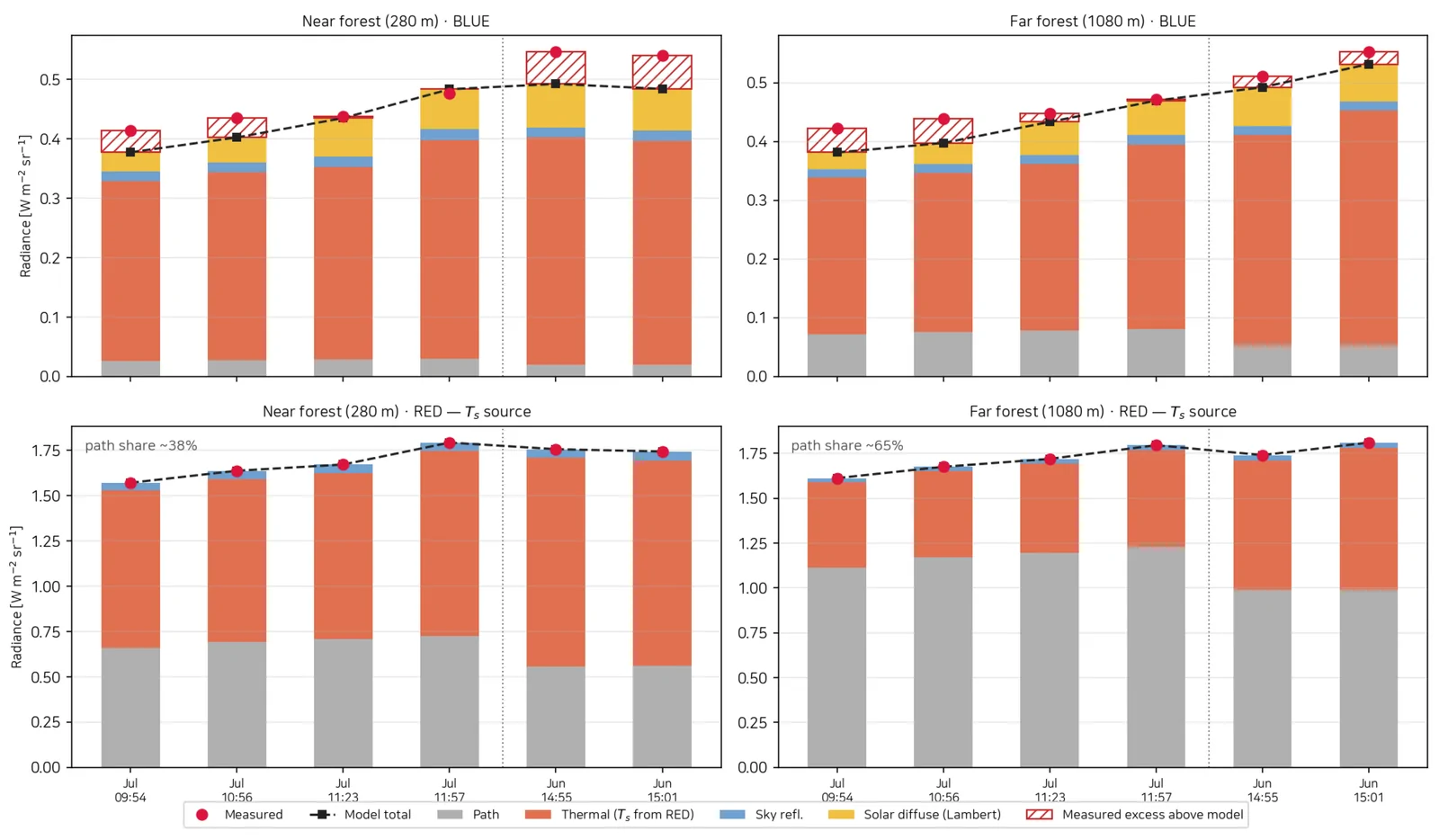
Forest radiance decomposition by band. Columns show the near (280 m) and far (1080 m) forests; rows show BLUE and RED. Surface temperature is retrieved from RED and held fixed for the BLUE prediction. Bars represent predicted components, black lines their sum, red dots the measurements, and hatching the measured excess above the model including Lambertian solar reflection. Mean RED path fractions across the six frames are 38% and 65%, respectively.

**Figure 9:**
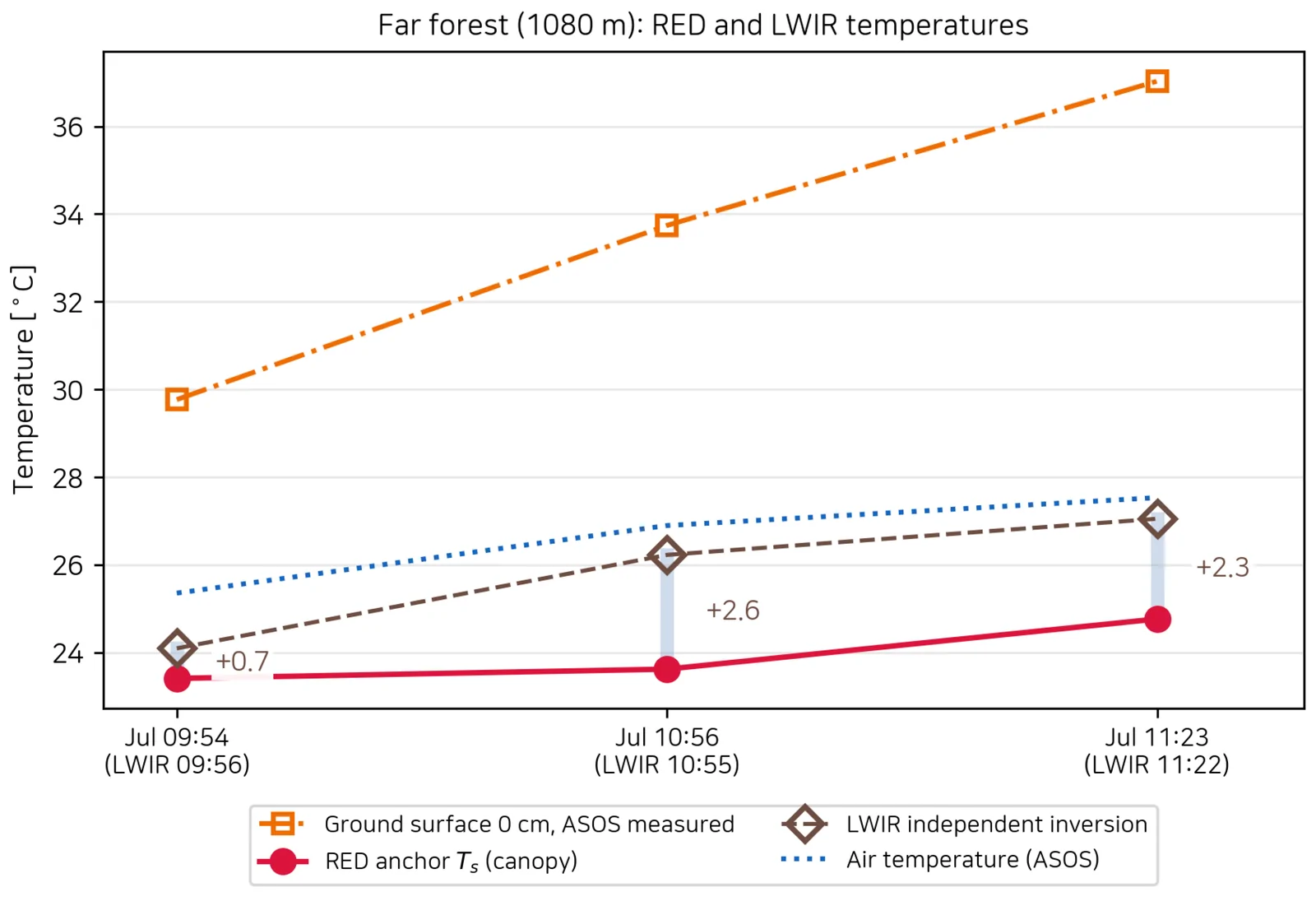
RED and independently retrieved LWIR temperatures for the far forest (1080 m) from three paired July observations. The LWIR–RED differences are +0.7, +2.6, and +2.3 °C, respectively. Air temperature and ASOS ground temperature at 0 cm are shown for environmental context.

**Figure 10:**
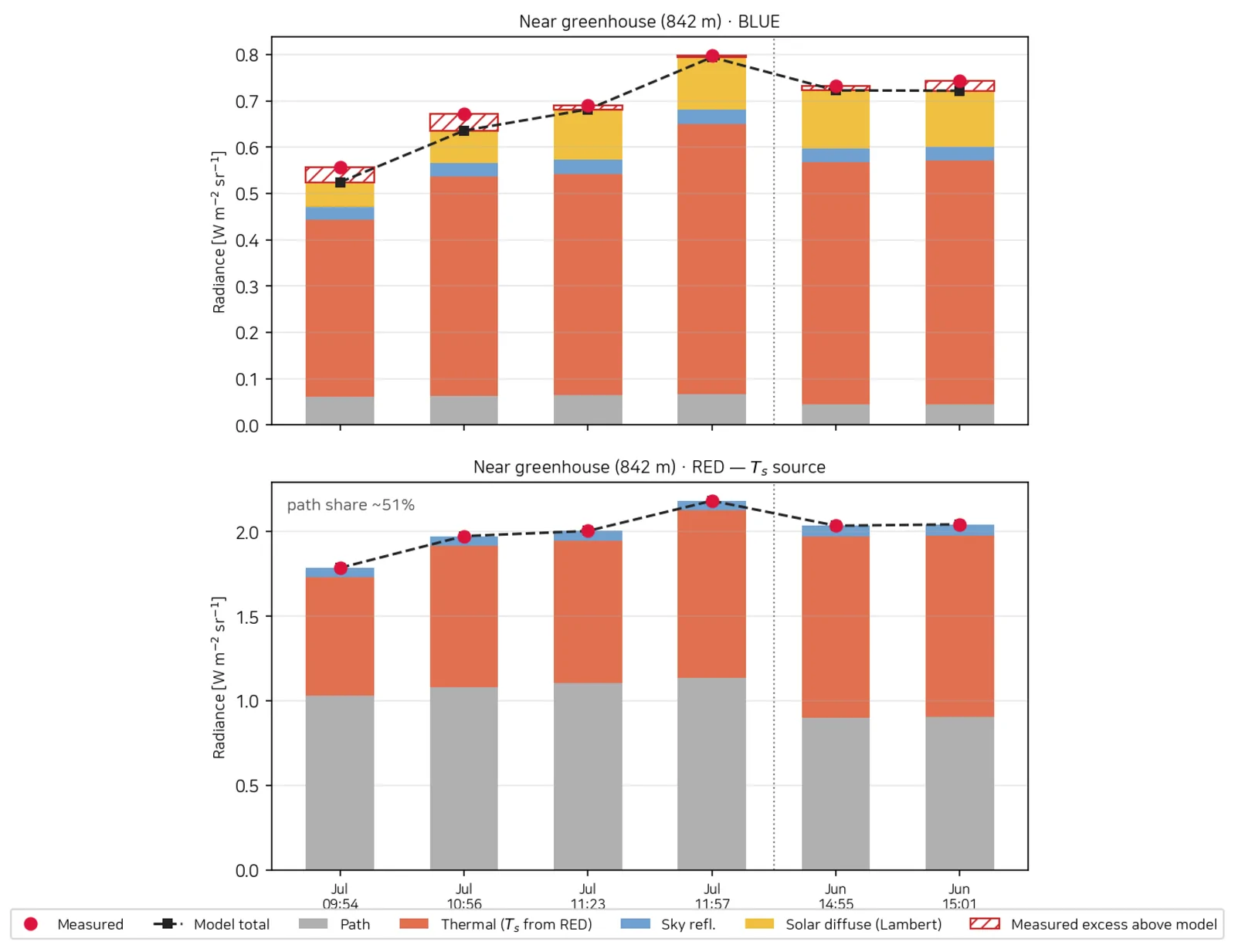
Radiance decomposition for the near greenhouse film at 842 m, using the format of Figure 8. With Lambertian diffuse solar reflection included, the maximum absolute BLUE residual across the six observations without glint is 0.037 W·m^−2^·sr^−1^ under the baseline calculation conditions.

**Figure 11:**
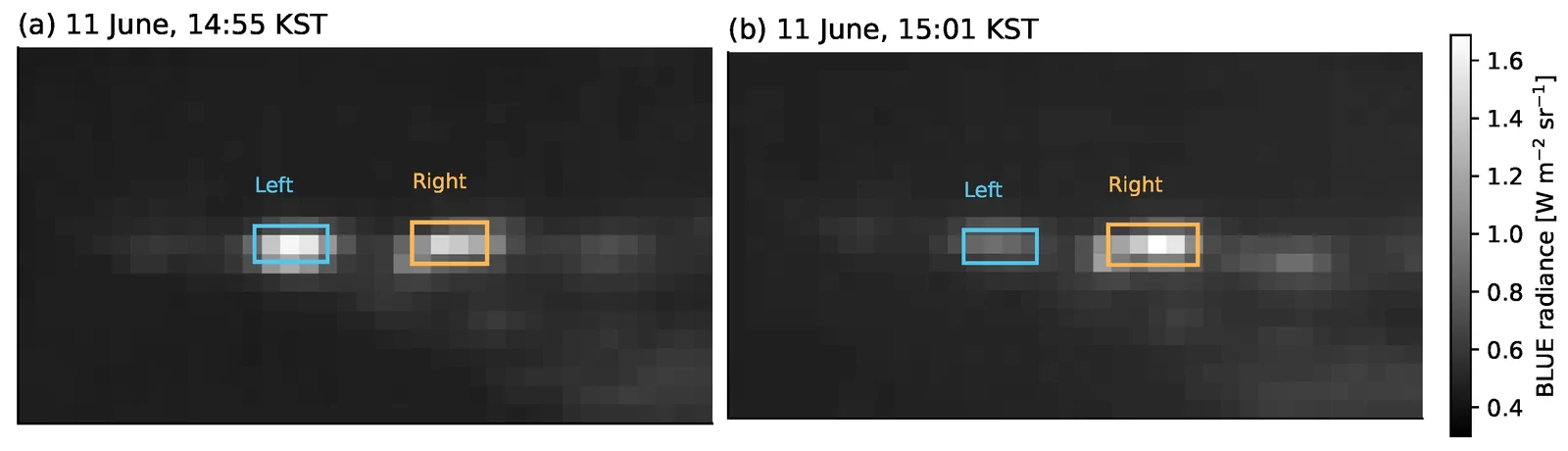
BLUE images of the distant polyethylene (PE) greenhouses on a common radiance scale: (**a**) 11 June at 14:55 and (**b**) 15:01 KST. Boxes mark the left- and right-building analysis regions. The left-building brightening decreases while the right remains bright.

**Figure 12:**
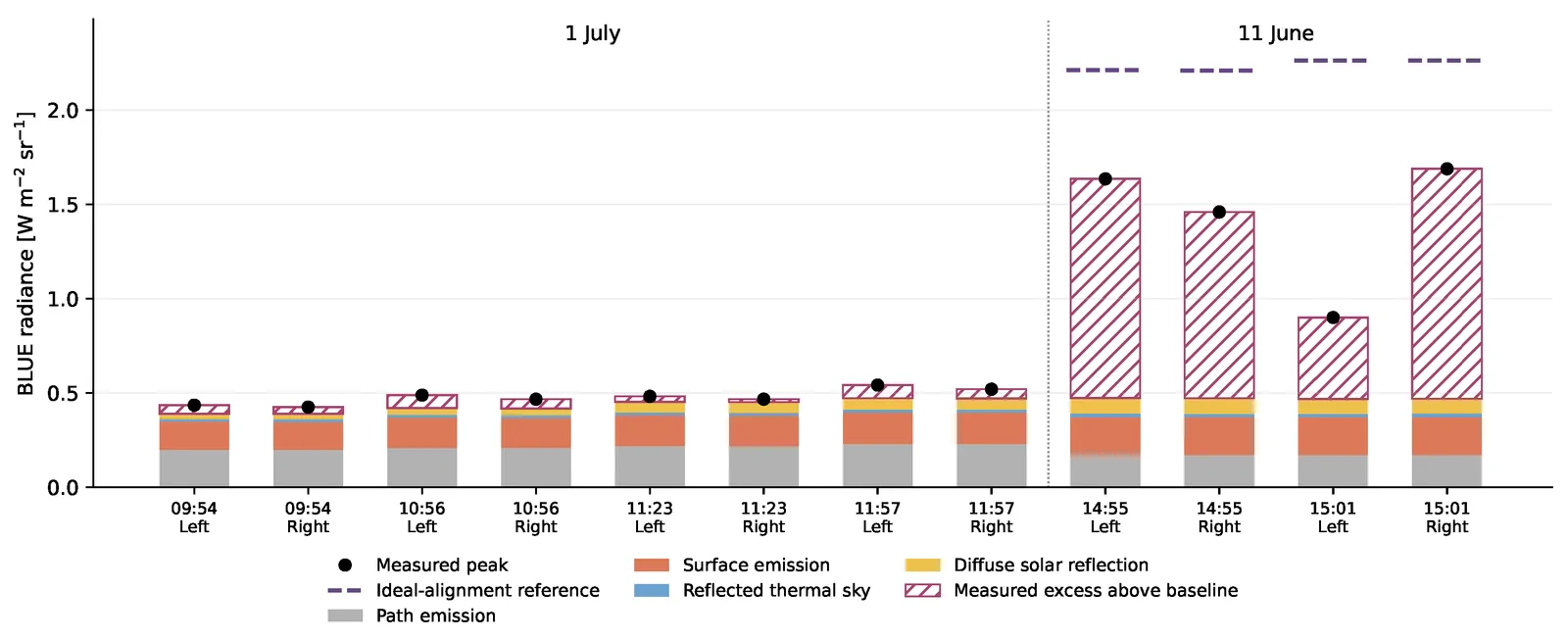
Peak-radiance comparison for both distant greenhouses. Colored bars show the baseline thermal, reflected-sky, and diffuse-solar components; hatching denotes measured excess above that baseline for both dates. Black dots are measured peaks. Purple dashed lines add the ideal-alignment reference from Equation (15) to the June baseline, using the adopted cylinder and pixel geometry.

**Table 1:** Instrument specifications and field acquisition settings. The MWIR effective field of view and nominal IFOV follow the image-based geometry analysis in Section 5.1; the LWIR field of view and IFOV use the specified angular coverage. LWIR frames were recorded at 30 Hz. MWIR: mid-wave infrared; LWIR: long-wave infrared; HFOV: horizontal field of view; IFOV: instantaneous field of view.

Item	Dual-Band MWIR (Primary)	LWIR (Auxiliary)
Detector	cooled photon detector	uncooled microbolometer
Array	512×512	640×480
Pixel pitch	12.2 μm	12 μm
Effective HFOV	100.3°	22.8°
IFOV	3.84 mrad (0.220°/px)	0.62 mrad (0.036°/px)
Integration time	1.2, 1.6, or 1.8 ms	fixed

**Table 2:** Ground-target recording conditions. Meteorological observations are from Icheon ASOS and irradiance ratios from Suwon ASOS. ASOS: Automated Synoptic Observing System; KST: Korea Standard Time. Solar positions use the listed times; sky-frame times are given in Section 5.2.

Date	Time	$T_{\mathrm{air}}$	RH	Cloud	Vis.	Sun		Irrad. Ratio
	(KST)	[°C]	[%]	[/10]	[km]	${\mathrm{az}}_{s}$ [°]	${\mathrm{el}}_{s}$ [°]	Meas./Clear
1 July 2026	09:54	25.4	71	10.0	33	100.7	52.8	0.56
	10:56	26.9	68	9.1	37	116.6	64.5	0.57
	11:23	27.5	66	8.2	36	127.3	69.0	0.85
	11:57	28.3	63	7.1	35	147.2	73.7	0.85
11 June 2026	14:55	25.8	47	7.0	49	255.7	56.1	0.97
	15:01	25.9	47	7.0	50	257.0	54.9	0.95

RH: relative humidity; Vis.: visibility. The irradiance ratio is measured global irradiance divided by its clear-sky prediction. June irradiance ratios remained close to unity despite a reported cloud amount of 7/10. Paired LWIR observations are available for the first three July sessions.

**Table 3:** Radiometric calibration coefficients for each channel using the models in Section 3.1. BLUE and RED coefficients were fitted to blackbody measurements in their respective sensitivity modes. *L* is band radiance, *t* is integration time, and *n* is the number of fitted measurements.

Sensor	Radiance Resp.	Dark Current	Bias	$R^{2}$	Resid. RMS	*n*
	C or $C^{\prime }$	$C_{\mathrm{DC}}$	$C_{0}$ or b		[DN]	
Dual-band BLUE	1328.8 *^a^*	2244.7 *^b^*	307.3 *^c^*	0.99996	6.4	48
Dual-band RED	1745.9 *^a^*	1271.5 *^b^*	165.8 *^c^*	0.99996	13.0	48
LWIR	28.998 *^d^*	—	6236.7 *^c^*	0.99963	5.6	8

*^a^* DN ms^−1^/(W m^−2^ sr^−1^). *^b^* DN ms^−1^. *^c^* DN. *^d^* DN/(W m^−2^ sr^−1^). With no integration-time variable, $C^{\prime }$ carries no ms^−1^ and cannot be compared directly with *C*.

**Table 4:** Viewing angles and adopted ranges obtained from the image-, satellite-, and terrain-based geometry analysis (Section 3.2). A dagger (^†^) identifies an attitude reference rather than a radiance target. Range sensitivity was assessed using ±25 m perturbations based on the DEM grid spacing; results are provided in Supplementary Materials, Section S4.3.

Target	az [°]	el [°]	Range [m]
Forest (near)	226.3	−13.2	280
Parking tower ^†^	220.6	−10.6	388
Greenhouse (near)	216.4	−6.9	842
Forest (far)	214.5	−5.5	1080
Art center ^†^	246.9	−2.8	1200
Greenhouse (far, L)	209.0	−1.8	5500
Greenhouse (far, R)	210.8	−1.8	5500

## Data Availability

The field imagery and calibration data were acquired under a defense research program and are available from the corresponding author upon reasonable request, subject to security review.

## Supplementary Materials

The following supporting information can be downloaded at: https://www.mdpi.com/article/10.3390/s26216959/s1. Sections S1–S6 provide band-integrated quantities, observation-specific comparisons, and sensitivity analyses. Figure S1: Molecular transmittance for the target paths; Figure S2: BLUE component changes under emissivity and aerosol perturbations; Figure S3: Film-emissivity effects and compensation between sky reflection and surface emission; Figure S4: Measured and predicted greenhouse radiances under the cloud scenarios; Figure S5: Spectral attenuation and its effect on the total BLUE signal; Table S1: Band-integrated blackbody radiance at reference temperatures; Table S2: Band-integrated solar-disc radiance; Table S3: Band-averaged transmittance for representative target paths; Table S4: Forest BLUE observations, predictions, and residuals by time; Table S5: Comparison metrics and evaluation scope; Table S6: Changes in RED temperature with solar reflection and emissivity variation; Table S7: Adopted inputs, sources, and sensitivity ranges; Table S8: Effects of varying the fixed emissivity inputs; Table S9: Sensitivity to a ±25 m range perturbation; Table S10: Cloud observations and baseline irradiance ratios corresponding to the image times; Table S11: Cloud-scenario parameter ranges and supporting references; Table S12: Observed-minus-predicted radiance residuals for the near greenhouse; Table S13: Comparison with and without the adopted glare term. References [24,32,33,34,35,36,37,38,39] are cited in the Supplementary Materials.
