# Supplementary material for: Model-Based Decomposition of Low-Altitude Infrared Radiance Using Dual-Band MWIR Observations
Source: Sensors (Basel). 2026 Nov 4;26(21):6959. doi: 10.3390/s26216959 (PMC13766441; doi:10.3390/s26216959)
Supplement: Supplementary file 1 [file sensors-26-06959-s001.zip › sensors-4609129-supplementary.pdf]

Supplementary Materials

Model-Based Decomposition of Low-Altitude Infrared Radiance Using Dual-Band MWIR Observations

S1 Band-integrated radiances and target-path transmittance

The shorter-wavelength band, BLUE, spans 3.5–4.2 μm, and the longer-wavelength band, RED, spans 4.4–5.2 μm. Band integration uses a rectangular spectral-response approximation, with a uniform response within each analysis band and zero response outside it, rather than a measured spectral-response curve. Tables S1 and S2 give the band-integrated Planck radiances used to characterize thermal and solar contributions. Figure S1 and Table S3 show the contrast in target-path attenuation for the atmospheric inputs used on the two observation days.

Table S1: Band-integrated blackbody radiance at reference temperatures.

Blackbody temperature (°C)	BLUE ($W \cdot m^{-2} \cdot sr^{-1}$)	RED ($W \cdot m^{-2} \cdot sr^{-1}$)	RED/BLUE
0	0.120890	0.655054	5.419
25	0.372755	1.628678	4.369
50	0.967979	3.522328	3.639

Planck radiance integrated over each analysis band at the stated temperature, before surface emissivity and path attenuation are applied.

Table S2: Band-integrated solar-disc radiance.

Solar-disc model	BLUE ($W \cdot m^{-2} \cdot sr^{-1}$)	RED ($W \cdot m^{-2} \cdot sr^{-1}$)	RED/BLUE
5800 K Planck radiance	111463.16	56381.37	0.5058

Band integrals of the 5800 K Planck model used for solar-disc radiance in the specular-reflection calculation. Sun-to-surface attenuation, surface reflection, and surface-to-camera attenuation are applied separately.

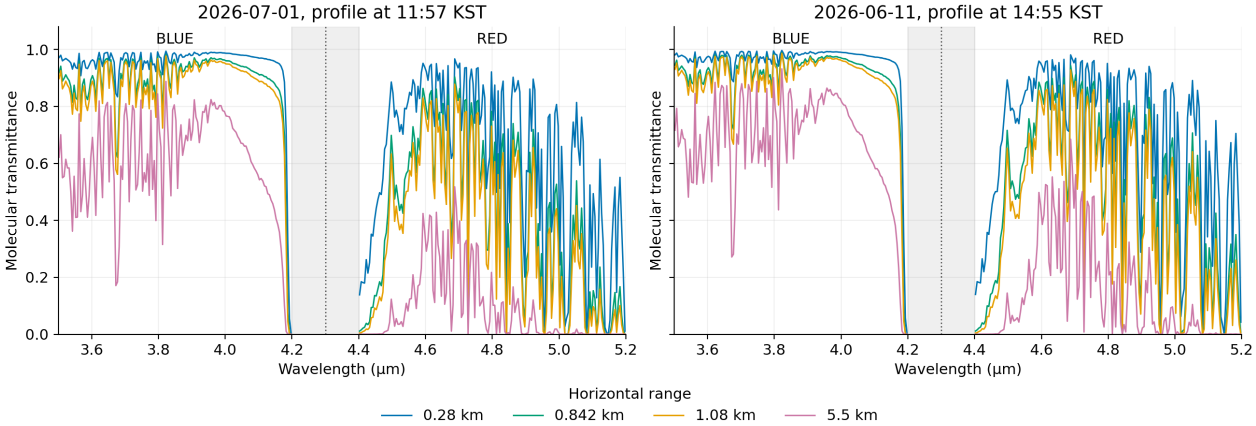

Figure S1: **Molecular transmittance for the target paths.** Line-By-Line Radiative Transfer Model (LBLRTM) calculations use the atmospheric inputs for 11:57 on 1 July 2026 and 14:55 on 11 June 2026. Ranges correspond to the near forest (280 m), near greenhouse (approximately 842 m), far forest (1080 m), and distant greenhouses (5500 m). Curves are averaged over 0.005 μm bins for display; band averages use the high-resolution spectra. Grey shading marks the interval between the analysis bands, and the dashed line marks the CO_2 absorption feature. Times are Korea Standard Time (KST).

Table S3: Band-averaged transmittance for representative target paths.

Observation date	Range (m)	BLUE, molecular	RED, molecular	BLUE, with aerosol	RED, with aerosol
2026-07-01	280	0.9464	0.6095	0.9311	0.6006
2026-07-01	842	0.8853	0.3936	0.8430	0.3767
2026-07-01	1080	0.8633	0.3433	0.8108	0.3245
2026-07-01	5500	0.6029	0.0821	0.4381	0.0617
2026-06-11	280	0.9554	0.6714	0.9479	0.6667
2026-06-11	842	0.9077	0.4715	0.8864	0.4616
2026-06-11	1080	0.8902	0.4217	0.8635	0.4104
2026-06-11	5500	0.6707	0.1315	0.5745	0.1145

Dimensionless band-averaged transmittances for the same atmospheric inputs as Figure S1. Molecular-only attenuation and attenuation including aerosols are listed separately.

S2 Observation-specific comparisons and error metrics

Residuals are defined as observed minus predicted radiance. Table S4 lists all 12 forest comparisons; their maximum absolute residual and root-mean-square (RMS) error are 0.0557 and 0.0329 $W·m^{-2}·sr^{-1}$, respectively. Table S5 identifies the observations and metric used for each comparison.

Table S4: Forest BLUE observations, predictions, and residuals by time.

Time	Near: observed	Near: predicted	Near: residual	Far: observed	Far: predicted	Far: residual
1 July 09:54	0.4144	0.3776	+0.0368	0.4227	0.3816	+0.0411
1 July 10:56	0.4358	0.4026	+0.0332	0.4386	0.3977	+0.0409
1 July 11:23	0.4385	0.4353	+0.0033	0.4484	0.4336	+0.0149
1 July 11:57	0.4765	0.4838	-0.0073	0.4715	0.4698	+0.0017
11 June 14:55	0.5471	0.4928	+0.0543	0.5114	0.4926	+0.0188
11 June 15:01	0.5401	0.4844	+0.0557	0.5533	0.5321	+0.0212

Units: $W·m^{-2}·sr^{-1}$. Near and far forests are at 280 and 1080 m. Residuals and summary statistics are calculated before rounding.

Table S5: Comparison metrics and evaluation scope. DN: digital numbers; LWIR: long-wave infrared; RMS: root mean square.

Comparison	Evaluation scope	Metric	Result
Forest BLUE	6 times × 2 ranges; 12 observations	Maximum absolute residual / RMS	0.0557 / 0.0329 $W·m^{-2}·sr^{-1}$
Sky BLUE	6 times; correction references excluded	RMS by observation time	0.0072–0.0202 $W·m^{-2}·sr^{-1}$ (13.5–24.5 DN)
Sky RED	6 times; correction references excluded	RMS by observation time	0.0027–0.0274 $W·m^{-2}·sr^{-1}$ (7.5–76.6 DN)
RED standard-atmosphere comparison	July 11:23 and June 15:01; same offsets	Standard-atmosphere RMS / observation-constrained RMS	10.93 / 15.40
Forest LWIR–RED	3 paired July observations	Temperature difference	+0.685, +2.603, +2.284°C
Greenhouse BLUE	6 times; baseline correction	Maximum absolute residual	0.0369 $W·m^{-2}·sr^{-1}$
Greenhouse cloud sensitivity	4 July times; spectral-attenuation scenarios using reported cloud cover	Maximum absolute residual	0.0619 $W·m^{-2}·sr^{-1}$

Sky RMS excludes the observation used for offset correction. The reference elevation is 6°, except at 14:55 on 11 June, when it is 10°. The standard-atmosphere comparison retains the same offset across atmospheric profiles within each image and evaluates the 10–30° observations. Cloud-scenario results are described in Section S5.

S3 Effect of RED solar reflection on temperature retrieval

To assess the effect of omitting RED solar reflection under strong solar illumination, we calculated RED solar irradiance without cloud attenuation using the atmospheric inputs and solar position for each observation time. The calculation used the low-resolution transmission (LOWTRAN) setting in libRadtran [1]. The resulting reflected-solar contribution was separated from the observed RED radiance before retrieving temperature again, and the new temperature was used in the BLUE thermal-emission prediction.

The largest temperature decrease was 0.243°C and the largest BLUE prediction change was 8.74 digital numbers (DN) (0.00375 $W \cdot m^{-2} \cdot sr^{-1}$). The forest maximum absolute BLUE residual changed from 0.0557 to 0.0594 $W \cdot m^{-2} \cdot sr^{-1}$, and RMS from 0.0329 to 0.0351 $W \cdot m^{-2} \cdot sr^{-1}$. Table S6 reports the observation-specific temperature changes, alongside the separate RED-emissivity test described in Section S4.1.

Table S6: Changes in RED temperature with solar reflection and emissivity variation.

Time	Forest range	Baseline RED temperature	ΔT with solar reflection	ΔT from emissivity variation
07-01 09:54	280 m	23.06	-0.140	-0.065∼+0.119
07-01 09:54	1080 m	23.42	-0.139	-0.073∼+0.135
07-01 10:56	280 m	24.06	-0.193	-0.052∼+0.096
07-01 10:56	1080 m	23.63	-0.196	-0.041∼+0.076
07-01 11:23	280 m	24.68	-0.203	-0.052∼+0.096
07-01 11:23	1080 m	24.77	-0.202	-0.055∼+0.101
07-01 11:57	280 m	27.83	-0.196	-0.111∼+0.204
07-01 11:57	1080 m	27.32	-0.199	-0.098∼+0.181
06-11 14:55	280 m	28.32	-0.243	-0.178∼+0.326
06-11 14:55	1080 m	28.98	-0.239	-0.192∼+0.352
06-11 15:01	280 m	27.94	-0.240	-0.168∼+0.308
06-11 15:01	1080 m	31.74	-0.218	-0.249∼+0.456

Temperatures are in °C; ΔT is relative to the baseline temperature in each row. Solar reflection uses irradiance calculated without cloud attenuation. Solar reflection and RED emissivity were evaluated separately. The emissivity range is the source-dataset minimum to maximum, 0.9044–0.9750.

S4 Sensitivity to surface, atmosphere, and range inputs

S4.1 Forest emissivity and aerosol optical depth

Forest-emissivity tests use the minimum and maximum band values among the 76 Quercus samples from the ECOsystem Spaceborne Thermal Radiometer Experiment on Space Station (ECOSTRESS) spectral library underlying the adopted medians [2]. The minimum sample has BLUE/RED values of 0.8967/0.9044 and the maximum has 0.9725/0.9750. Each sample's two band values are applied together to retrieve RED temperature again and recalculate BLUE thermal emission and reflection.

These emissivity changes produce RED temperature changes of −0.249 to +0.456°C and BLUE prediction changes of −83.2 to +154.0 DN. The largest component change occurs in solar reflection, through the emissivity-dependent reflectance $1-\varepsilon$ (Figure S2a).

Aerosol optical depth is multiplied by 0.8, 1.0, or 1.2 while retaining aerosol type and spectral shape. Changes in surface solar irradiance and atmospheric transmission and thermal emission along the target-to-camera path are propagated through RED temperature retrieval and BLUE prediction. The RED temperature changes by less than 0.042°C and the predicted ground-target BLUE signal by less than 1.7 DN under these perturbations.

Figure S2b illustrates the partial cancellation between changes in surface and path emission. For the far forest at 11:57 in July, reducing aerosol optical depth by 20% increases BLUE surface emission by 8.83 DN and decreases path emission by 9.29 DN; including reflection gives a net increase of 1.65 DN. DN changes use the integration time and BLUE calibration response of each observation.

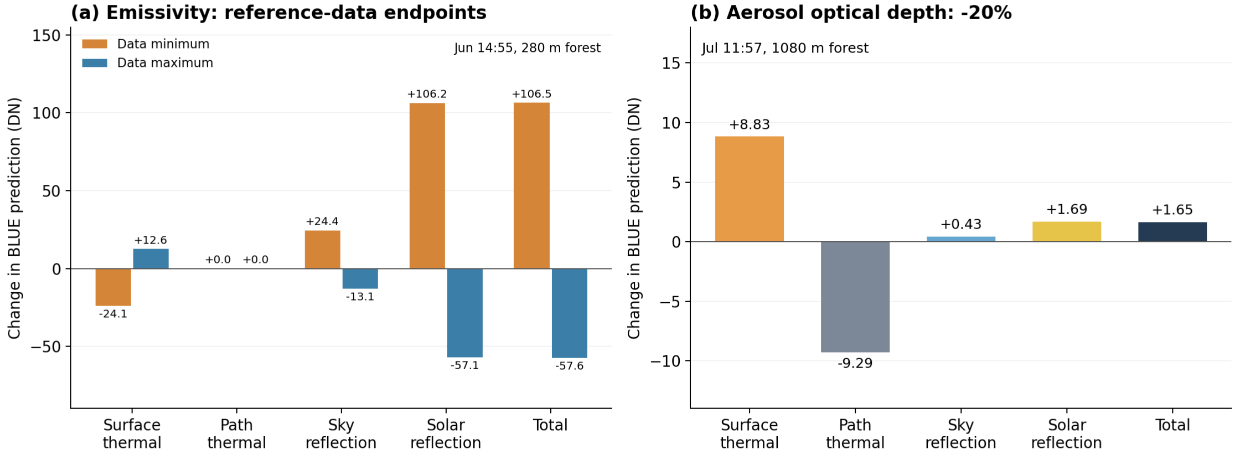

Figure S2: **BLUE component changes under emissivity and aerosol perturbations.** (a) Minimum and maximum data-derived emissivity pairs for the near forest at 14:55 in June. (b) A 20% reduction in aerosol optical depth for the far forest at 11:57 in July. Surface emission increases by 8.83 DN while path emission decreases by 9.29 DN; including reflected components gives a net increase of 1.65 DN. Bars show changes from baseline and their sum. DN: digital numbers.

S4.2 Effective film and sky emissivities

Table S7 summarizes the adopted values and test ranges. Film emissivity is varied from 0.85 to 0.95 and effective sky emissivity from 0.75 to 0.95, one at a time. Temperature is retrieved again from RED for every condition. Film emissivity controls the reflected solar contribution through $1-\varepsilon$. The thermal sky input is approximated as $\varepsilon_{\text{sky}}B_k(T_{\text{air}})$; surface reflectance and target-path transmittance are applied to obtain its reflected contribution at the camera. For the sky input, changes in reflected radiation are partly offset by changes in surface thermal emission after temperature retrieval (Figure S3).

Table S7: Adopted inputs, sources, and sensitivity ranges.

Input	Adopted value	Basis	Sensitivity range
Forest BLUE/RED	0.946 / 0.949	Median band values from 76 ECOSTRESS Quercus samples: 0.9458 / 0.9485.	Minimum sample: BLUE 0.8967 / RED 0.9044; maximum sample: BLUE 0.9725 / RED 0.9750
Near greenhouse	0.90	Effective value adopted with reference to PE optical constants reported by David et al. (2022)[3], allowing for possible soiling and ageing	0.85–0.95
Reflected-sky input	0.85	Common value in a greybody approximation informed by the air-temperature/effective-emissivity representation of Brutsaert (1975)[4]	0.75–0.95

The forest range uses paired band values from individual library samples. Film and sky ranges assess variation around the common effective values adopted for the observations.

Table S8: Effects of varying the fixed emissivity inputs.

Input varied	Target	Temperature change from baseline (°C)	Change in total BLUE prediction (DN)	BLUE observed-minus-predicted residual range ($W \cdot m^{-2} \cdot sr^{-1}$)
Effective film emissivity 0.85–0.95	Greenhouse; 6 times	−0.846∼+0.926	−149.23∼+149.46	−0.05485∼+0.08415
Effective sky emissivity 0.75–0.95	Forest; 2 ranges × 6 times	−0.178∼+0.177	−0.94∼+0.94	−0.00761∼+0.05607
Effective sky emissivity 0.75–0.95	Greenhouse; 6 times	−0.277∼+0.276	−3.03∼+3.05	+0.00220∼+0.03807

Each input is changed separately, with RED temperature retrieved again. DN changes use the corresponding integration time and BLUE calibration response. Temperature and prediction changes are relative to baseline; residual ranges are observed minus predicted radiance.

Across the tested observations, sky-emissivity variation changes BLUE predictions by at most 0.94 DN for forests and 3.05 DN for the greenhouse. Film-emissivity variation produces larger changes, reaching approximately 149.5 DN. The greenhouse agreement at the adopted film emissivity of 0.90 therefore depends more strongly on the surface-reflection input than on the effective sky emissivity within these ranges.

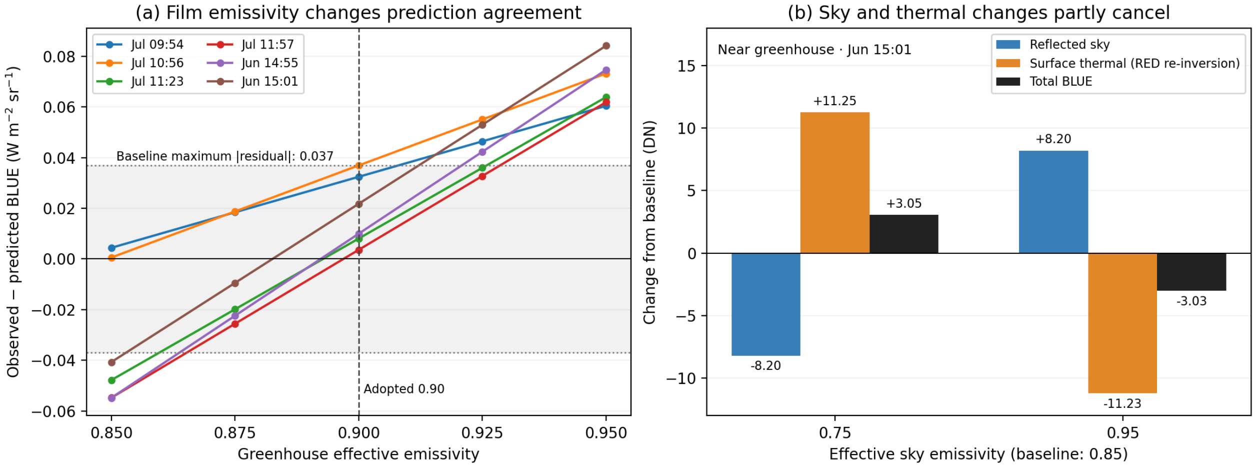

Figure S3: **Film-emissivity effects and compensation between sky reflection and surface emission.** (a) Greenhouse BLUE residuals at six times. The vertical dashed line marks the adopted film emissivity of 0.90. The grey interval (± 0.037 W·m⁻²·sr⁻¹) is the maximum absolute residual across those six observations at the adopted value. (b) Component changes for the greenhouse at 15:01 in June when effective sky emissivity is changed from 0.85 to 0.75 or 0.95. Repeating the RED temperature retrieval changes BLUE surface emission, partly offsetting the change in reflected sky radiance.

S4.3 Target range

Target ranges are perturbed by ± 25 m, corresponding to the DEM grid spacing. Path transmittance and RED temperature are recalculated before predicting BLUE. This is a range-sensitivity test; the perturbation is not an independently measured range uncertainty.

Table S9: Sensitivity to a ±25 m range perturbation.

Target	Adopted range (m)	Range perturbation (m)	RED temperature change (°C)	BLUE prediction change (W·m⁻²·sr⁻¹)
Far forest	1080	±25	-0.061∼+0.061	-0.000622∼+0.000625
Near forest	280	±25	-0.088∼+0.089	-0.001283∼+0.001309
Near greenhouse	842.5	±25	-0.207∼+0.206	-0.002898∼+0.002891

Changes span the six observation times. The computational greenhouse range is 842.5 m, described as approximately 842 m in the manuscript.

S5 Spectral effects of cloud attenuation

The baseline calculation already uses measured global irradiation to account for reduced illumination. The additional analysis evaluates the sensitivity of applying the same relative reduction to BLUE by calculating wavelength-dependent attenuation for the four July greenhouse observations. Detailed cloud records from automated weather observations at Gimpo Airport, the nearest such records available to us, define the cloud types and mixing fractions. The baseline irradiance ratios use Suwon Automated Synoptic Observing System (ASOS) measurements divided by the corresponding clear-sky calculations (Table S10). Cloud optical depth and particle size are varied over the grids in Table S11 to assess spectral sensitivity.

Table S10: Cloud observations and baseline irradiance ratios corresponding to the image times.

Image time, 1 July	Gimpo observation time	Cirrus cover	Other reported cloud type and cover	Baseline irradiance ratio from Suwon
09:54	10:00	7/8	Altocumulus 5/8	0.56
10:56	11:00	6/8	Altocumulus 4/8	0.57
11:23	11:00	6/8	Altocumulus 4/8	0.85
11:57	12:00	6/8	Stratocumulus 1/8	0.85

Korea Meteorological Administration (KMA) Gimpo Airport weather observations and Suwon Automated Synoptic Observing System (ASOS) records [5]. Cloud cover is reported for each layer. Suwon hourly accumulated solar irradiation is converted to mean irradiance before comparison with the clear-sky model.

Table S11: Cloud-scenario parameter ranges and supporting references.

Cloud type	Optical depth τ_{550}	Effective particle radius (µm)	References used to select the range
Cirrus	0.03, 0.1, 0.2, 0.3, 0.5, 1	15, 30, 50	Observed mean optical depth 0.30±0.36 and particle-size sensitivity: [6], [7]
Altocumulus	0.3, 1, 3, 6, 10	6, 10, 16	Mean optical depths 6.40 and 9.28; effective radii 8.76 and 14.03 µm in two cloud populations: [8]
Stratocumulus	0.3, 1, 3, 5, 10, 20	6, 10, 16	Range extending to thinner and thicker conditions around a reported optical depth of 8.6: [9]

τ_{550} is vertical extinction optical depth at 550 nm. Optical depth and effective particle radius are varied independently within each grid; the references inform the selected ranges.

Cirrus is represented by ice particles and the other two types by liquid droplets, using the same libRadtran discrete ordinate radiative transfer (DISORT) solver as the sky-radiance and surface-irradiance calculations in §3.3 [1]. Each cloud type is calculated separately: cirrus at all four times, altocumulus at the first three, and stratocumulus at 11:57. Cloud-base altitudes are approximately 6.114, 2.761, and 1.542 km above sea level (ASL), respectively; assumed layer thicknesses are 1.0, 0.5, and 0.5 km. Atmospheric inputs and solar position are held at their observation-specific values.

For cloud fraction f, surface irradiance is mixed as $E(f) = (1-f)E_{\text{clear}} + fE_{\text{cloudy}}$ separately in BLUE and the 0.3–2.8 µm broadband interval. Let r_B and r_p be the resulting cloudy-to-clear irradiance ratios. The factor $K = r_B/r_p$ multiplies only the baseline BLUE solar-reflection term. The original measured-irradiance adjustment, RED temperature, and all other radiance components are retained. The reported cloud fractions define the weighted cases; $f = 1$ defines a separate fully covered comparison addressing the overcast conditions noted by the reviewer. These scenarios assess the spectral approximation without replacing the baseline with an inferred cloud state.

Applying the spectral attenuation difference in scenarios using the reported cloud fractions lowers total predicted BLUE radiance by 0.08–5.70% relative to baseline. The maximum residual in this sensitivity assessment is 0.0619 W·m^{-2}·sr^{-1} compared with the baseline value of 0.0369 W·m^{-2}·sr^{-1}, but remains smaller at every time than the residual obtained by excluding solar reflection (Table S12). Thus the calculated agreement varies with the cloud assumptions, while inclusion of diffuse solar reflection continues to improve the prediction under the tested weighted conditions. Fully covered columns produce larger effects, with a maximum radiance decrease of 15.4% and maximum residual of 0.1165 W·m^{-2}·sr^{-1}.

Table S12: Observed-minus-predicted radiance residuals for the near greenhouse.

Time, 1 July	Baseline residual	Residual range, cloud-cover-weighted scenarios	Residual without solar reflection	Maximum residual, fully covered columns
09:54	0.0324	0.0329–0.0577	0.0867	0.0852
10:56	0.0369	0.0374–0.0619	0.1070	0.1045
11:23	0.0080	0.0088–0.0469	0.1173	0.1130
11:57	0.0036	0.0043–0.0301	0.1167	0.1165

Units: W·m^{-2}·sr^{-1}. Residuals are observed minus predicted radiance. Weighted ranges use Tables S10 and S11. The last column gives the maximum across optical-depth and particle-size combinations when each cloud type is evaluated separately at full cover.

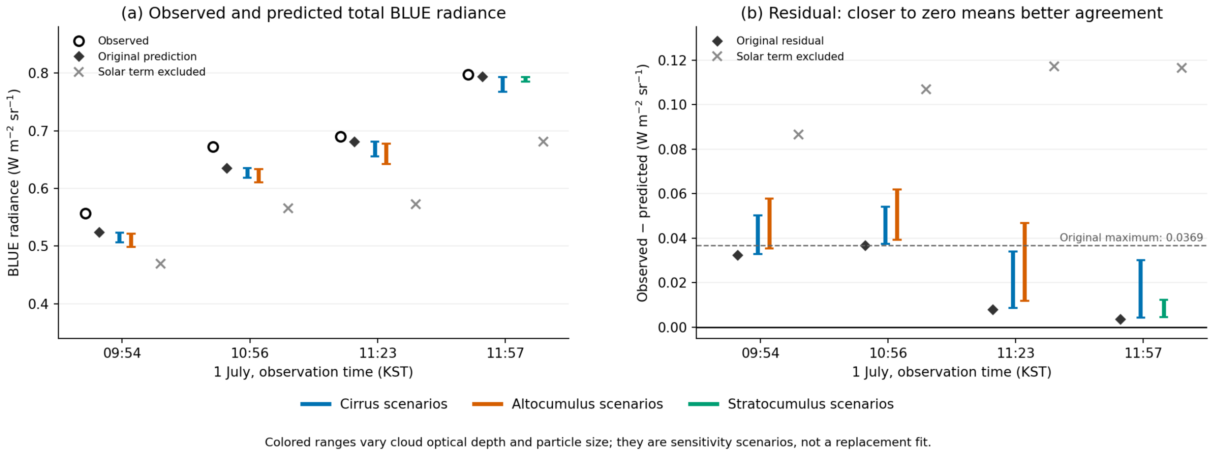

Figure S4: **Measured and predicted greenhouse radiances under the cloud scenarios.** (a) Measurements (open circles), baseline predictions (diamonds), cloud-cover-weighted ranges (colored intervals), and predictions without solar reflection (crosses). (b) Observed-minus-predicted residuals; values closer to zero indicate closer agreement. Intervals span the optical-depth and particle-size grids. The dashed line marks the baseline maximum residual of 0.0369 W·m^{-2}·sr^{-1}. Cloud types are calculated separately.

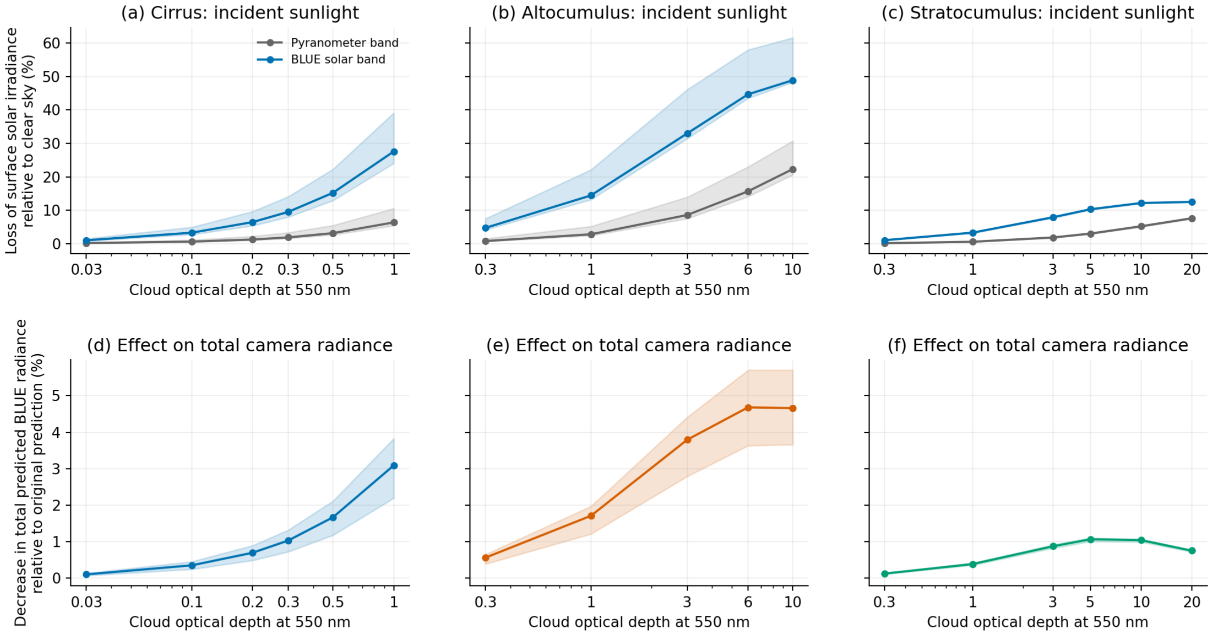

Figure S5: **Spectral attenuation and its effect on the total BLUE signal.** Upper panels (a–c) show reductions in broadband and BLUE surface solar irradiance relative to clear-sky conditions. Lower panels (d–f) show the reduction in total predicted BLUE radiance relative to baseline after applying K to the solar-reflection term. Lines show medians and shading spans particle sizes and observation times, with the reported cloud fractions used as mixing weights.

S6 Residual comparison with common glare coefficients

The adopted BLUE glare coefficients, $A = 1000$ and $n = 2.5$, are evaluated using 29 valid sky measurements at six observation times. The mean difference between observed and predicted DN is treated as a frame-specific additive offset before calculating brightness-temperature residuals. The resulting brightness-temperature residual RMS is 1.047°C with glare and 1.631°C without it, a reduction of approximately 36% (Table S13). This RMS reduction represents improved agreement with the BLUE sky observations used for coefficient selection. This additional comparison separates angular variation from a constant offset; the main sky results retain correction at the lowest valid elevation.

Table S13: Comparison with and without the adopted glare term.

Comparison	Sum of squared brightness-temperature residuals (°C^2)	Pooled RMS (°C)
Without glare	77.150	1.631
Adopted coefficients A=1000, n=2.5	31.819	1.047

The same coefficients are applied at all six times. The sum of squared residuals and pooled RMS use the 29 valid sky points after separating the mean DN offset within each frame.

References

[1] C. Emde, R. Buras-Schnell, A. Kylling, B. Mayer, et al. The libRadtran software package for radiative transfer calculations (version 2.0.1). *Geoscientific Model Development*, 9:1647–1672, 2016. https://doi.org/10.5194/gmd-9-1647-2016.

[2] Susan K. Meerdink, Simon J. Hook, Dar A. Roberts, and Elsa A. Abbott. The ECOSTRESS spectral library version 1.0. *Remote Sensing of Environment*, 230:111196, 2019. https://doi.org/10.1016/j.rse.2019.05.015.

[3] Mauro David, Davide Disnan, Anna Lardschneider, Dominik Wacht, Hanh T. Hoang, Georg Ramer, Hermann Detz, Bernhard Lendl, Ulrich Schmid, Gottfried Strasser, and Borislav Hinkov. Structure and mid-infrared optical properties of spin-coated polyethylene films developed for integrated photonics applications. *Optical Materials Express*, 12(6):2168, 2022. https://doi.org/10.1364/ome.458667.

[4] Wilfried Brutsaert. On a derivable formula for long-wave radiation from clear skies. *Water Resources Research*, 11(5):742–744, 1975. https://doi.org/10.1029/wr011i005p00742.

[5] Korea Meteorological Administration. Open Meteorological Data Portal: Gimpo Airport AMOS and Suwon ASOS observations. https://data.kma.go.kr/. Observations for 1 July 2026.

[6] Wei Wang, Fan Yi, Fuchao Liu, Yunpeng Zhang, Changming Yu, and Zhenping Yin. Characteristics and seasonal variations of cirrus clouds from polarization lidar observations at a 30°N plain site. *Remote Sensing*, 12(23):3998, 2020. https://doi.org/10.3390/rs12233998.

[7] Kevin Wolf, Nicolas Bellouin, and Olivier Boucher. Sensitivity of cirrus and contrail radiative effect on cloud microphysical and environmental parameters. *Atmospheric Chemistry and Physics*, 23(21):14003–14037, 2023. https://doi.org/10.5194/acp-23-14003-2023.

[8] Wencai Wang, Lifang Sheng, Hongchun Jin, and Yongqing Han. Dust aerosol effects on cirrus and altocumulus clouds in Northwest China. *Journal of Meteorological Research*, 29(5):793–805, 2015. https://doi.org/10.1007/s13351-015-4116-9.

[9] S. Ghosh, P. R. Jonas, and R. Wood. Some impact of pollutants on the development and optical properties of stratocumulus clouds. *Quarterly Journal of the Royal Meteorological Society*, 126(569):2851–2872, 2000. https://doi.org/10.1002/qj.49712656912.
